# Mutant Best1 Expression and Impaired Phagocytosis in an iPSC Model of Autosomal Recessive Bestrophinopathy

**DOI:** 10.1038/s41598-018-21651-z

**Published:** 2018-03-14

**Authors:** Alan D. Marmorstein, Adiv A. Johnson, Lori A. Bachman, Cynthia Andrews-Pfannkoch, Travis Knudsen, Benjamin J. Gilles, Matthew Hill, Jarel K. Gandhi, Lihua Y. Marmorstein, Jose S. Pulido

**Affiliations:** Department of Ophthalmology, Mayo Clinic, 200 First Street SW, Rochester, MN 55905 USA

## Abstract

Autosomal recessive bestrophinopathy (ARB) is caused by mutations in the gene *BEST1* which encodes bestrophin 1 (Best1), an anion channel expressed in retinal pigment epithelial (RPE) cells. It has been hypothesized that ARB represents the human null phenotype for *BEST1* and that this occurs due to nonsense mediated decay (NMD). To test this hypothesis, we generated induced pluripotent stem cells (iPSCs) from a patient with ARB and her parents. After differentiation to retinal pigment epithelial (iPSC-RPE) cells, both *BEST1* mRNA and Best1 protein expression were compared to controls. *BEST1* mRNA expression levels, determined by quantitative PCR, were similar in ARB iPSC-RPE, parental cells, and genetically unrelated controls. Western blotting revealed that CRALBP and RPE65 were expressed within the range delineated by unrelated controls in iPSC-RPE from the ARB donor and her parents. Best1 protein was detected in different clones of ARB iPSC-RPE, but at reduced levels compared to all controls. When tested for the ability to phagocytose photoreceptor outer segments, ARB iPSC-RPE exhibited impaired internalization. These data suggest that impaired phagocytosis is a trait common to the bestrophinopathies. Furthermore, ARB is not universally the result of NMD and ARB, in this patient, is not due to the absence of Best1.

## Introduction

Mutations in the gene *BEST1* cause five clinically recognized retinal degenerative eye diseases in man: Best vitelliform macular dystrophy (BVMD)^1,2^, adult onset vitelliform macular dystrophy (AVMD)^3^, autosomal recessive bestrophinopathy (ARB)^4^, autosomal dominant vitreoretinochoroidopathy (ADVIRC)^5^, and retinitis pigmentosa 50 (RP50)^6,7^. These diseases are collectively referred to as the bestrophinopathies. The most common of the bestrophinopathies is BVMD, an autosomal dominant macular degeneration affecting as many as 1 in 18,000 individuals in the United States^8^. BVMD, AVMD, RP50, and ADVIRC all exhibit a dominant pattern of inheritance. ARB and some instances of RP50^6^ exhibit an autosomal recessive inheritance. Clinical abnormalities associated with mutations in *BEST1* are limited to the eye^9^. ADVIRC and RP50 are peripheral retinopathies, though macular cone function can be affected in ADVIRC^6,10^ and we have observed cystoid macular edema in RP50^7^. Fluid- and debris-filled retinal detachments in the macula appear to be common to AVMD, BVMD, and ARB.

ARB, in contrast to BVMD, typically exhibits a juvenile age of onset with disease symptoms presenting in the first decade^9^. In ARB, the serous retinal detachment observed is typically much broader than the vitelliform lesion common to BVMD and is normally flanked by small yellow spots. Smaller peripheral lesions may also be present^9^. Optical coherence tomography (OCT) images from patients with BVMD and ARB are both characterized by elongated photoreceptor outer segments (OS) that are uneven in length as well as piles of debris in the sub-retinal space^11,12^. Studies performed in our laboratory using *Best1*^*W93C*^ knock-in mice, a mouse model of BVMD, demonstrate that the debris is comprised primarily of unphagocytosed OS^13^.

*BEST1* encodes bestrophin 1 (Best1), an integral membrane protein^14^ that functions as an anion channel that is regulated by both Ca^2+^ ^15–17^ and volume^18,19^. Best1 also functions as a regulator of intracellular Ca^2+^ signaling^13,17,20,21^ and exerts effects on store-operated calcium entry^22^ as well as voltage-dependent calcium channels^23–25^. Although most studies have assessed Best1’s ability to mediate chloride transport, Best1 has been reported to be highly permeable to a variety of other anions as well, including bicarbonate^26^. Strong evidence exists that Best2, a structurally similar paralog of Best1, functions as a bicarbonate channel *in vivo*^27–29^. In the eye, Best1 is uniquely expressed by RPE cells where it is predominantly localized to the basolateral plasma membrane^14^. Recently, the crystal structure was solved for bacterial^30^ and chicken^31^ Best1, demonstrating that this evolutionarily conserved protein forms homo-pentameric oligomers with an ion conductance pore at its center^30,31^.

ARB has been hypothesized to represent the human null phenotype for *BEST1*^4^. This idea originates from the identification of ARB patients homozygous or compound heterozygous for truncating mutations (http://www-huge.uni-regensburg.de/BEST1_database/home.php?select_db=BEST1). Evidence for this hypothesis comes predominantly from two different studies^32,33^. The first by Davidson *et al*.^32^ identified altered pre-mRNA splicing using an *in vitro* assay of a novel ARB mutation. They predicted that this splicing variant would lead to a premature termination codon that would be degraded by NMD^32^. The second study by Pomares *et al*.^33^ collected blood total RNA from two different ARB patients and performed RT-PCR on these blood samples. The authors reported that most of the mutated *BEST1* transcripts were eliminated before translation and this was attributed to NMD^33^. A third line of evidence arguing for the “null” phenotype hypothesis comes from canine multifocal retinopathy (cmr), another animal model of bestrophinopathy^9^. Dogs with cmr exhibit a retinal phenotype^34–36^ that is comprised of multifocal lesions that individually resemble those found in BVMD, rather than the broad serous detachments observed in humans with ARB^9^. Analysis of postmortem eyes from dogs with cmr suggests that Best1 is absent in the RPE^37^.

Data from mice, however, differ. Mice lacking Best1 exhibit no retinal disease phenotype^19,21^ and instead show abnormally high intracellular calcium levels following retinal stimulation with ATP as well as an enhanced dc-ERG response^21^. In contrast, mice harboring the BVMD mutation W93C exhibit a phenotype highly reminiscent of BVMD^13^. Recent work by Uggenti *et al*.^38^, which examined expression of ARB-associated Best1 mutants in MDCK cells, suggests that protein misfolding may be a key contributor to the pathogenesis of ARB rather than NMD. Moreover, ARB mutations have been identified that do not appear to impact anion channel activity or expression *in vitro*^39,40^, further indicating the pathogenesis is more complex than a complete lack of expression.

In the quest to reconcile these apparently disparate data sets, understanding whether ARB is in fact a null phenotype seems a critical question. In this study, we generated induced pluripotent stem cells (iPSCs) from a pediatric patient with ARB. We then differentiated these stem cells into RPE and sought to determine whether ARB is a null phenotype by examining iPSC-RPE from a donor with ARB compound heterozygous for the mutations R141H and I366fsX18 in *BEST1*. We further sought to perform disease modeling to better understand the pathogenesis of ARB. Our findings suggest that NMD does not contribute to the pathogenesis of ARB in this patient. Instead, we find that these mutant forms of Best1 are expressed, albeit at low protein levels, and lead to an impaired ability to phagocytose photoreceptor OS. These data leave us to hypothesize that ARB is either due to a Best1 protein insufficiency or due to dysfunction induced by expressed ARB mutant protein.

## Results

### Case summary

**Figure 1:**
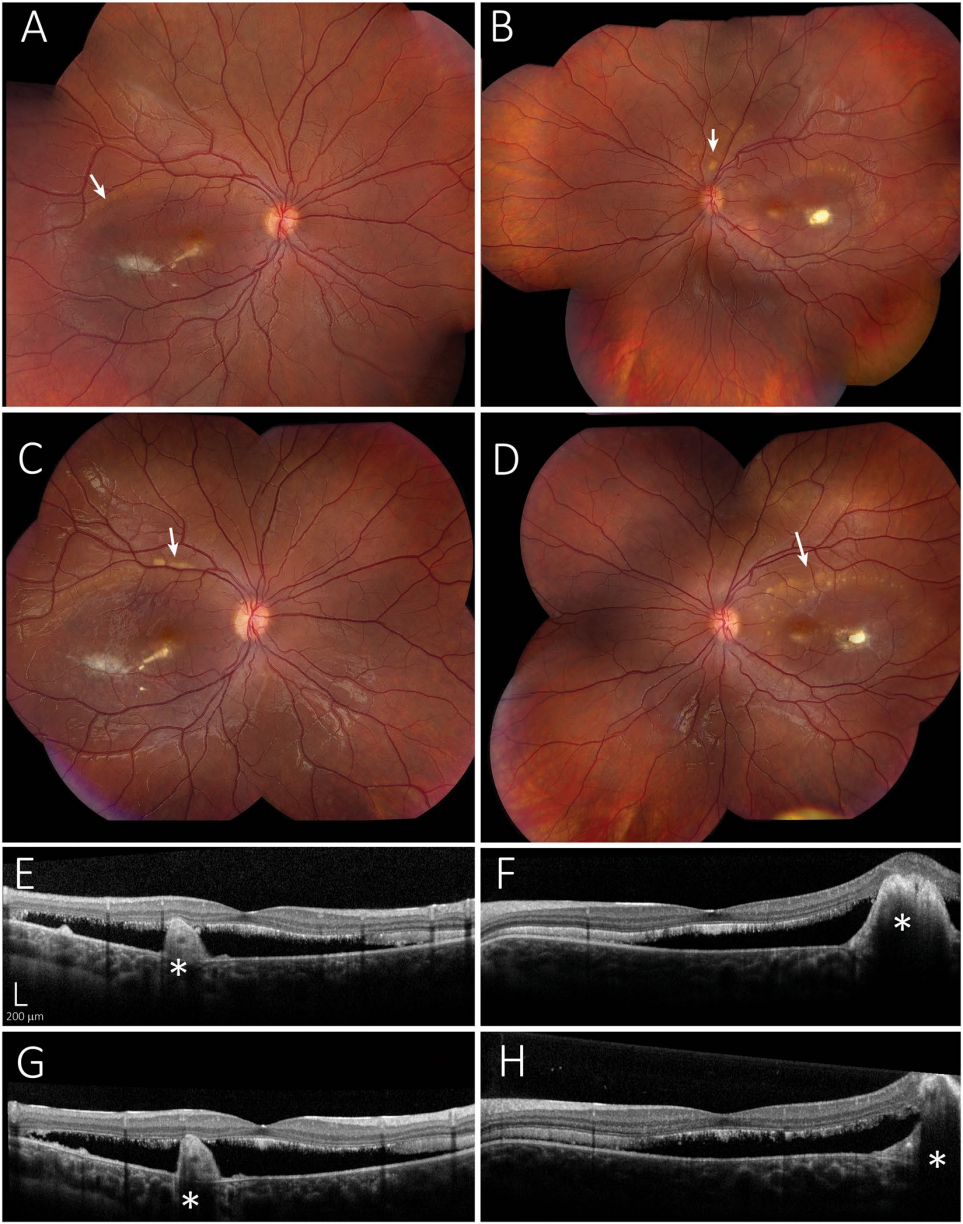
Comparison of fundus photographs of patient with ARB over a five year period. Right eye fundus in 2012 (**A**) has a central scar with surrounding subretinal fluid and multiple small barely visible vitelliform lesions (arrows) just within the superotemporal arcade. There are now larger vitelliform lesions that have actually extended out of the macular region into the superotemporal arcade in the right eye fundus in 2017 (**C**). Fundus photograph of the left eye of the patient in 2012 (**B**) shows a central scar with surrounding subretinal fluid and multiple small visible vitelliform lesions just within the superotemporal arcade (arrows). There are also vitelliform lesions superior to the disc. In 2017 (**D**) the lesions superior to the disc are not as visible and there are more vitelliform lesions in the superior macula delineating the superior edge of the subretinal fluid (arrow). OCT imaging of the right eye in 2012 (**E**) shows elongated photoreceptor OS or “stalactites”, subretinal debris, and a large mound indicated by * and corresponding to the central scar. In 2017, retinal topology has remained similar, but there is some thinning of the stalactites indicating loss of photoreceptors. The left eye has shown small but similar changes between 2012 (**F**) and 2017 (**H**). Scale bar in E applies to the OCT images in F, G, and H.

We previously reported a case of a young Caucasian female diagnosed with ARB^39^. The subject was referred to our clinic at the age of seven and has been followed routinely ever since. She is now 16 years of age. Genetic testing detected compound heterozygous mutations in *BEST1*. These mutations were R141H (CGC > CAC) and a novel, frameshift mutation I366fsX18 (c.1098_1100 + 7del)^39^. Both clinical genetic testing and NextGen™ DNA sequencing revealed that the subject’s father is a heterozygous carrier for the mutation R141H (CGC > CAC) and that the mother is a heterozygous carrier for the mutation I366fsX18 (c.1098_1100 + 7del). No familial history of ophthalmic disease was indicated and neither parent presented with an abnormal fundus. As of her last visit in August 2017, the subject’s visual acuity remained stable at 20/50 (OD) and 20/30 (OS). In 2012 (Fig. 1A,B), the subject exhibited subretinal fibrosis, bilateral macular lesions, and apparent multifocal sub-RPE lesions in both the right (Fig. 1C) and left (Fig. 1D) eyes. Though the extent of subretinal fluid observed has varied, her fundus appearance has remained remarkably stable with only minor remodeling of the vitelliform lesions noted over the last five years (Fig. 1). OCT images for both eyes remain comparable between 2012 (Fig. 1E,F) and 2017 (Fig. 1G,H). Abnormally elongated photoreceptor OS or “stalactites”, subretinal fluid, and subretinal debris are apparent from OCT imaging (Fig. 1E–H). Skin biopsies were obtained from the subject and her parents under informed consent as part of a larger study on bestrophinopathies conducted at the Mayo Clinic (NCT02162953, https://clinicaltrials.gov/ct2/show/NCT02162953). Fibroblasts obtained from the skin biopsies were reprogrammed to iPSCs and further differentiated into RPE cells.

### Differentiation and characterization of iPSC-RPE

To test the hypothesis that ARB is the human “null” phenotype, we sought to model ARB *in vitro* using iPSC-RPE derived from the subject. Controls for our experiments were iPSC-RPE from three unaffected, unrelated donors and iPSC-RPE from the subject’s mother and father (Table 1). One of those lines, IMR-90, clone 4, is an established iPSC line that has been characterized elsewhere^41^. The successful generation of iPSCs from all donors was confirmed by the presence of the pluripotency markers Oct4, SSeA4, Nanog, Tra1–60, and Sox1^39^. For iPSCs derived from the subject and her parents, multiple clones were used in this study. Four iPSC clones were generated from the father. Two of those clones exhibited an abnormal karyotype. The remaining two clones (Dad147 and Dad150) produced iPSC-RPE with yields for Dad147 exceeding those of Dad150. Due to differences in iPSC-RPE yield, the majority of our studies were performed using Dad147 and validated by comparison with Dad150. Both the subject’s and her mother’s fibroblasts were reprogrammed twice, yielding four clones for each reprogramming event. For the mother, one clone (Mom201) produced iPSC-RPE from the initial reprogramming and a single clone (Mom214) produced iPSC-RPE from the second reprogramming. Mom201 produced very low yields of iPSC-RPE and were used primarily to validate results obtained from Mom214. For the subject with ARB, two reprogramming events were performed. From the first, a single clone (ARB243) produced small amounts of iPSC-RPE cells. From the second reprogramming we were able to produce iPSC-RPE from two of four clones (ARB17 and ARB226). ARB17 produced only small amounts of iPSC-RPE while ARB226 generated the higher yield of cells. As such, ARB226 became the line used in all experiments with ARB243 and ARB17 being used to confirm results from ARB226. Table 1 summarizes the different iPSC-RPE lines used and their origins.

**Table 1:** Cell lines used in this study.

iPSC line	Source*	Clone #(s)	Alias	Age	Gender	BEST1 Genotype	Relationship to ARB Subject
006-BIOTR-0001	MCB	1	Control 1	21	Female	+/+	Unrelated
IMR-90	WC	4	Control 4	n/a†	Female	+/+	Unrelated
001-BIOTR-0001	MCB	138	Control 138	80	Female	+/+	Unrelated
011-BIOTR-0001	MCB	147, 150	Dad	37	Male	+/R141H	Father
011-BIOTR-0002	MCB	201, 214,	Mom	34	Female	+/I366fsX18	Mother
011-BIOTR-0003	MCB	17, 226, 243	ARB	13	Female	R141H/I366fsX18	Subject

^*^MCB = Mayo Clinic BioTrust (Rochester, MN); WC = WiCell (Madison, WI).

^†^IMR-90 was reprogrammed from an immortalized human fibroblast cell line generated from a 20 week gestation age fetus. IMR90 cells were reprogrammed using retroviral vectors.

**Figure 2:**
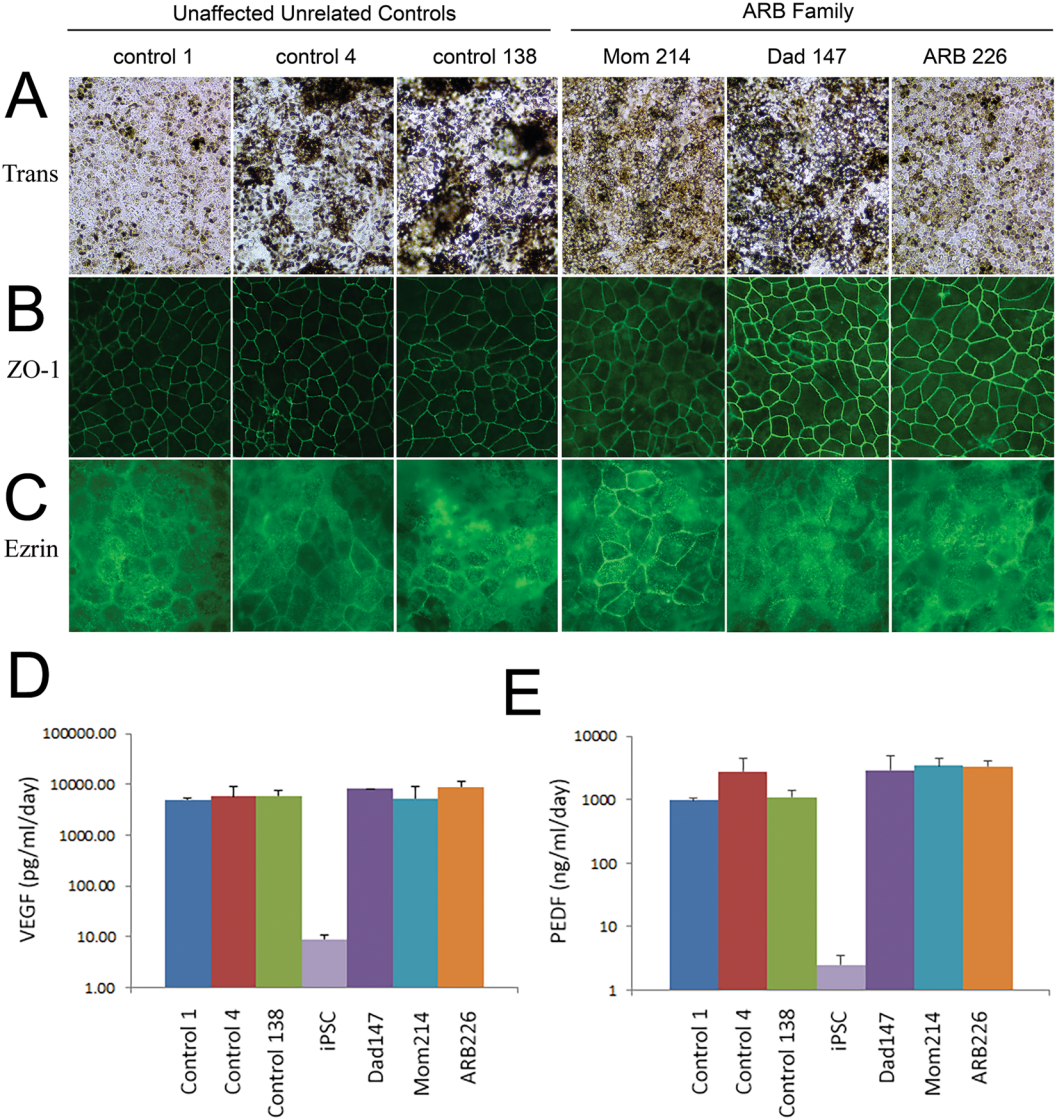
Characterization of iPSC-RPE clones. iPSC-RPE were generated from a pediatric patient with ARB, her parents, and three unrelated, unaffected controls. Each iPSC-RPE line is marked by a descriptor which defines the original donor as well as a number which defines the clone number used. (**A**) Transmitted light images of these iPSC-RPE reveal dense pigmentation and a cobble-stone-like appearance that is classical of RPE. (**B**) Immunofluorescence of ZO-1, a tight junction marker, shows that iPSC-RPE from each donor formed robust tight junctions. (**C**) These iPSC-RPE lines generated significant apical microvilli, as indicated by immunofluorescent staining of the RPE polarity marker ezrin. (**D**) Compared to a negative control (iPSCs alone), each iPSC-RPE displayed notable secretion of VEGF. (**E**) Each iPSC-RPE line also exhibited dramatically increased levels of PEDF secretion compared to iPSCs.

**Figure 3:**
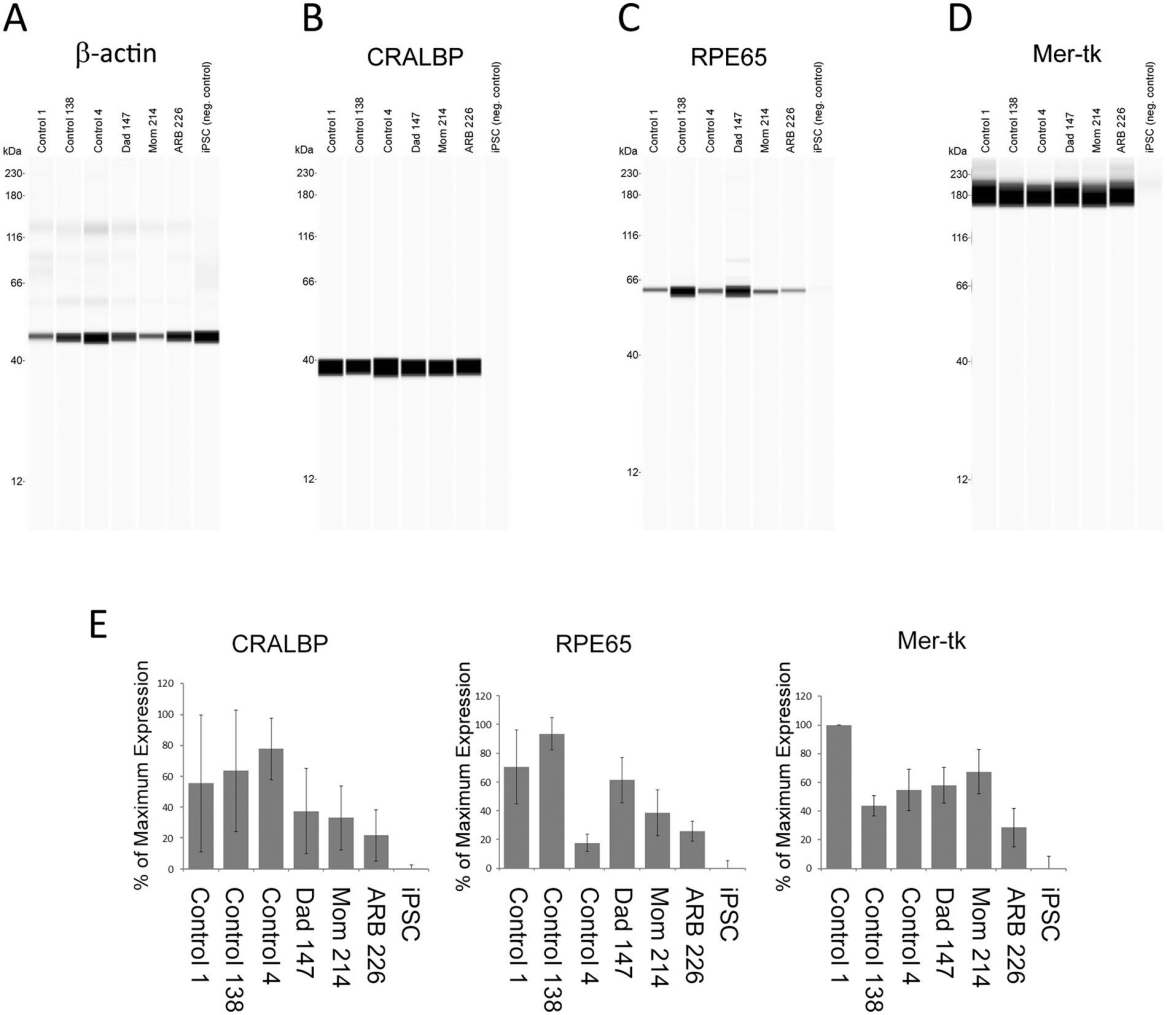
iPSC-RPE from all donors express RPE marker proteins. (**A**) As a loading control, each iPSC-RPE line (ARB patient, the patient’s parents, and three unrelated, unaffected controls) as well as iPSCs (negative control) were blotted for ß-actin. (**B**) Unlike iPSCs, all iPSC-RPE lines showed robust expression of CRALBP. (**C**) Just like CRALBP, iPSC-RPE from the patient, the patient’s mom, the patient’s dad, and the three unrelated, unaffected controls all expressed RPE65. The negative control (iPSCs) had no detectable RPE65 protein expression. (**D**) Each of the iPSC-RPE lines but not iPSCs displayed strong expression of the RPE marker Mer-tk. (**E**) The relative expression levels for each of these three markers is summarized for iPSCs and all of the iPSC-RPE lines. Graphs show the summary of expression for each marker from three separate blotting experiments. Variability between each of the controls as well as between the different iPSC-RPE lines was observed for each of the RPE markers. Control 4 displayed the highest levels of CRALBP, Control 138 had the highest levels of RPE65, and Control 1 showed the highest levels of Mer-tk.

All iPSC lines and clones used produced confluent monolayers of cells with abundant pigment granules and exhibited the classic hexagonal, cobblestone appearance typical of RPE cells (Fig. 2A). Examination of gene expression by reverse transcription PCR using a panel of 36 markers (Table 2), including RPE-specific markers as well as pluripotency markers, revealed that 100% of clones of control iPSC-RPE lines used were positive for all RPE markers and negative for the pluripotency marker Lin28A. All iPSC-RPE lines were also negative for Sendai virus and “Yamanaka” factors introduced by reprogramming, although some continued to express endogenous NANOG (Table 2). Immunofluorescent staining of iPSC-RPE cells with an anti-ZO-1 antibody confirmed monolayers produced cell-cell junctions (Fig. 2B). Along with the robust, continuous circumferential staining of ZO-1 (Fig. 2B), the formation of tight junctions and functionally intact monolayers was demonstrated by the transepithelial electrical resistance (TER) of the different iPSC-RPE lines grown on permeable supports (Table 3). Although there was variability in TER among donor lines and to a lesser degree among clones derived from the same donor (Table 3), the TER values obtained are typical of iPSC-RPE. More specifically, they are within the range of values we have previously observed for Control 1^39^, consistent with the findings of others for iPSC-RPE monolayers^42–44^, and consistent with the values observed for RPE *in vivo*^44–46^. Anti-ezrin antibody staining confirmed that abundant apical microvilli were expressed at the surface of every clone (Fig. 2C). Examination of the media from the cells indicated that all iPSC-RPE secreted abundant VEGF (Fig. 2D) and PEDF (Fig. 2E). With ß-actin used as a loading control (Fig. 3A), Western blots demonstrated expression of the cellular RPE markers CRALBP (Fig. 3B), RPE65 (Fig. 3C), and Mer-tk (Fig. 3D) in all iPSC-RPE lines. The relative expression of CRALBP, RPE65, and Mer-tk is summarized in Fig. 3E. This summarized data indicates the expression level averaged from three separate blotting experiments. While each iPSC-RPE line expressed all RPE markers examined, there was line-to-line variability in expression between the unrelated controls, the iPSC-RPE from the ARB patient, and the iPSC-RPE from the patient’s parents (Fig. 3E).

**Table 2:** iPSC-RPE Gene Expression Analysis by RT-PCR.

Gene	Ctrl. 1	Ctrl. 4	Ctrl. 138	ARB 226	ARB 243	Mom 201	Mom 214	Mom 221	Dad 147	Amplicon Size	Forward Primer	Reverse Primer
RPE65	+	+	+	+	+	+	+	+	+	101	AACTTGGGTTTGGCAAGAGC	CCACACTCAGAACTACACCATCA
5′ BEST1	+	+	+	+	+	+	+	+	+	359	ATTTATAGGCTGGCCCTCACGGAA	TGTTCTGCCGGAGTCATAAAGCCT
3′ BEST1	+	+	+	+	+	+	+	+	+	125	TGCCAGAGATCCCCGAAAAT	GGAATGTGCTTCATCCCTGTT
MERTK	+	+	+	+	+	+	+	+	+	172	ACTGCCTGGATGAACTGTATGA	GAGCTCTCCAGCAACTGTGT
LRAT	+	+	+	+	+	+	+	+	+	277	TGGTCTCCAACAAGCGTCTC	TCACAAAACTTGTCGGACTGG
RDH5 (RDH)	+	+	+	+	+	+	+	+	+	259	CCTTCCTCACCAAGTACCTGAAA	CTCTTGCTGGAAGGCTGGAT
RLBP1 (CRALBP)	+	+	+	+	+	+	+	+	+	122	AAGCTGCTTGAGAGGGTCTTT	ACGGCCTTGCCATCATACTT
CRX	+	+	+	+	+	+	+	+	+	150	CCAGGGTTCAGGTTTGGTTC	CATCTGTGGAGGGTCTTGGG
MITF	+	+	+	+	+	+	+	+	+	142	AATACAGGAACTTGAAATGCAGGC	ATGCTGAAGGAGGTCTTGGC
TFE3	+	+	+	+	+	+	+	+	+	130	CGGGAGATCTCTGAGACCGA	GGATGAGAGTGCCCAGTTCC
TFEB	+	+	+	+	+	+	+	+	+	159	CGCATCAAGGAGTTGGGAAT	CTCCAGGCGGCGAGAGT
MLANA	+	+	+	+	+	+	+	+	+	181	CTGCTCATCGGCTGTTGGTA	GAGCATTGGGAACCACAGGT
PMEL	+	+	+	+	+	+	+	+	+	127	TGCCTGGGATTCTTCTCACAG	TGCTTCATAAGTCTGCGCCTAT
TYR	+	+	+	+	+	+	+	+	+	324	TTGACAGTATTTTTGAGCAGTGGC	GACACAGCAAGCTCACAAGC
GPR143 (OA1)	+	+	+	+	+	+	+	+	+	236	TCTGAAGGTTCTGATGCCAGC	GCTGGTGATGAGAGCAAGGT
OCLN	+	+	+	+	+	+	+	+	+	223	AAGCAAGTGAAGGGATCTGC	TCACAGAGGTTTGGCTTCCG
EZR	+	+	+	+	+	+	+	+	+	307	CGCTCTAAGGGTTCTGCTCT	TCCTGGGCAGACACCTTCTTA
CDH2	+	+	+	+	+	+	+	+	+	341	ATCCTGCTTATCCTTGTGCTGA	GGGTCATTGTCAGCCGCTTT
ITGB5	+	+	+	+	+	+	+	+	+	306	GGTGGACACCATCGTGAAAG	GAAGCCATTTCATAGCGGGC
ITGAV	+	+	+	+	+	+	+	+	+	124	AATGTCACCTGGGGCATTCA	AAAAGCCCATCCTGTACATTACAAA
SERPINF1 (PEDF)	+	+	+	+	+	+	+	+	+	349	CTTCAAGGGGCAGTGGGTAAC	GGACTTGGTGACTTCGCCTT
AQP11	+	+	+	+	+	+	+	+	+	110	TCCGAACCAAGCTTCGTATC	TAGCGAAAGTGCCAAAGCTG
AQP1	+	+	+	+	+	+	+	+	+	134	TGGACACCTCCTGGCTATTG	GGGCCAGGATGAAGTCGTAG
SLC16A8 (MCT3)	+	+	+	+	+	+	+	+	+	119	CTGCAGTTCGAGGTGCTCAT	AGGCGGCCGGCAGAG
SLC16A1 (MCT1)	+	+	+	+	+	+	+	+	+	242	ACCACTTTTAGGTCGGCTCA	TCTGGTCCGGAGATTCTGCT
CD147	+	+	+	+	+	+	+	+	+	129	AACTCTTCCTGAGGCAGGTGG	GGAATCTACGGGGTGGGTTT
HIF1A	+	+	+	+	+	+	+	+	+	128	GCCAGACGATCATGCAGCTA	GCAGTCTACATGCTAAATCAGAGG
OTX2	+	+	+	+	+	+	+	+	+	134	TCGAGGGTGCAGGTATGGTT	TCTGAACTCACTTCCCGAGC
NCAM	+	+	+	+	+	+	+	+	+	157	ACTGACGGAGCCCGAGAAG	TTGCTCGGTTCTCTTCACCC
CD36	+	+	+	+	+	+	+	+	+	125	TTGGCTTAATGAGACTGGGACC	ACATCACCACACCAACACTGA
ACTB	+	+	+	+	+	+	+	+	+	207	GATCAAGATCATTGCTCCTCCTG	CTGCGCAAGTTAGGTTTTGTCA
GAPDH	+	+	+	+	+	+	+	+	+	109	CTCTGCTCCTCCTGTTCGAC	ACCAAATCCGTTGACTCCGA
LIN28A	−	−	−	−	−	−	−	−	−	256	AGATCAAAAGGAGACAGGTGCT	AATAGCCCCCACCCATTGTG
NANOG	+	+	+	−	−	+	+	−	−	190	GTGACGCAGAAGGCCTCA	TGCACCAGGTCTGAGTGTTC
SeV*	−	−	−	−	-	−	−	−	−	181	GGATCACTAGGTGATATCGAGC	ACCAGACAAGAGTTTAAGAGATATGTATC
SeV KLF*	−	−	−	−	−	−	−	−	−	410	TTCCTGCATGCCAGAGGAGCCC	AATGTATCGAAGGTGCTCAA
SeV KOS*	−	−	−	−	−	−	−	−	−	528	ATGCACCGCTACGACGTGAGCGC	ACCTTGACAATCCTGATGTGG
SeV c-myc*	−	−	−	−	−	−	−	−	−	532	TAACTGACTAGCAGGCTTGTCG	TCCACATACAGTCCTGGATGATGATG

*SeV, SeV KLF, SeV KOS, and SeV c-myc are “Yamanaka” factors delivered using Sendai virus. The PCR primers are designed specifically to identify the Sendai delivered transgenes which are expected to be absent. The abbreviation “Ctrl.” stands for control.

**Table 3:** Transepithelial Electrical Resistances of iPSC-RPE after Eight Weeks in Culture.

iPSC line	Clone	Designation	TER* (Ohms* cm^2^)
006-BIOTR-0001	1	Control 1	132 ± 11
IMR90	4	Control 4	78 ± 8
001-BIOTR-0001	138	Control 138	nd^†^
011-BIOTR-0001	147	Dad147	185 ± 26
	150	Dad150	229 ± 18
011-BIOTR-0002	201	Mom201	85 ± 13
	214	Mom214	129 ± 14
011-BIOTR-0003	17	ARB17	nd^†^
	226	ARB226	185 ± 25
	243	ARB243	188 ± 14

^*^Average ± sd, n > 10.

^†^nd = not done.

### Is mutant Best1 subject to NMD?

**Figure 4:**
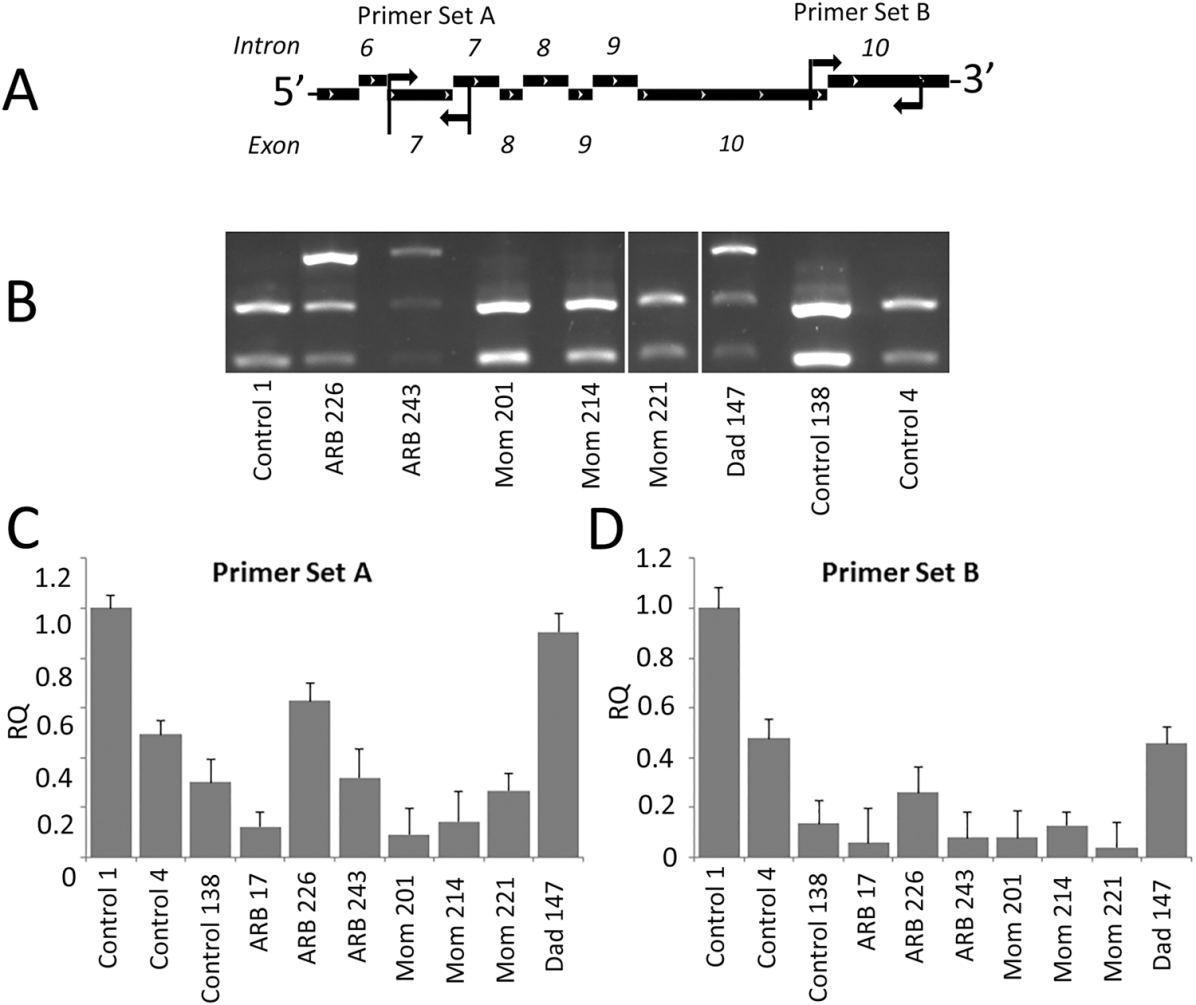
*BEST1* mRNA is expressed in iPSC-RPE from an ARB patient. (**A**) Two sets of PCR primers were generated which amplified a fragment of *BEST1* that spans the R141H mutation but abolishes an Fsp1 restriction site. PCR products were purified and digested with FspI. WT *BEST1* mRNA was cleaved into two fragments by FspI, but the R141H mutation was not susceptible to cleavage by this enzyme. (**B**) The result was that iPSC-RPE clones containing the R141H mutation (ARB 226, ARB 243, Dad 147) showed three mRNA fragments. All other clones either homozygous for WT *BEST1* (Control 1, Control 138, Control 4) or heterozygous with I366fsX18 and WT *BEST1* (Mom 201, Mom 214, Mom 221) showed only two mRNA fragments. *BEST1* was detectable in each clone, regardless of the patient origin. (**C**) Using Primer Set A, *BEST1* mRNA levels varied between the three unrelated, unaffected controls as well as between the ARB patient and her parents. Clone ARB 226 showed higher mRNA levels than the Control 4 and Control 138 lines. (**D**) Using Primer Set B, *BEST1* mRNA levels also varied between each of the iPSC-RPE lines. Clone ARB 226 still exhibited a greater amount of *BEST1* mRNA than Control 138.

In their original description of ARB, Burgess *et al*.^4^ suggested that ARB may be the “null phenotype” for *BEST1* in man. A few years later, Pomares *et al*.^33^ suggested that this “null phenotype” was due to NMD, a hypothesis that has gained some popularity^9^. Contrary to this hypothesis, our gene expression panel (Table 2) generated using reverse transcription PCR (RT-PCR) detected *BEST1* in all iPSC-RPE lines (Table 1), including all clones analyzed from the pediatric subject with ARB. However, RT-PCR can be exquisitely sensitive and detect minute quantities of mRNA. To determine if *BEST1* mRNA is subject to NMD in ARB, we performed quantitative PCR (qPCR) to compare levels of expression of *BEST1* mRNA between donors and clones. Two different primer sets spanning introns were used (Fig. 4A). Primer set A was designed to produce a product for WT *BEST1* mRNA as well as mRNA for *BEST1*^*R141H*^ and *BEST1*^*I366fsX18*^. Primer set B was designed to yield a product for WT *BEST1* and *BEST1*^*R141H*^ only. *BEST1* mRNA was expressed in every donor and clone examined (Fig. 4B) using both primer sets. Clones from the subject with ARB expressed *BEST1* mRNA at levels similar to parental controls (Fig. 4B), though there was variability in total expression between all clones for both primer set A (Fig. 4C) and primer set B (Fig. 4D). While *BEST1* mRNA in ARB226 and ARB243 was expressed at similar levels to those observed in the unrelated controls, ARB17 showed lower levels than each of the unrelated controls regardless of the primer set used. The variability observed between lines is likely due to clonal variation^47^.

To determine whether mRNA for both *BEST1* mutants was present in our ARB subject, we performed PCR using primers designed to amplify a fragment of Best1 cDNA spanning the R141H mutation which abolishes an FspI restriction site (TGCGCA > TGCACA). PCR products were purified and digested with FspI. WT *BEST1* mRNA was cleaved into two fragments by FspI, but the R141H mutation is not susceptible to cleavage by the enzyme. As shown in Fig. 4B, iPSC-RPE clones heterozygous for the R141H mutation have three mRNA fragments: the WT sequence cut into two bands and the uncut mutated sequence. Since ARB226 and ARB243 have three bands, similar to Dad147, we conclude that mRNA for both mutants is expressed and that ARB in this patient is not the result of NMD.

### Is Best1 protein expressed in ARB?

**Figure 5:**
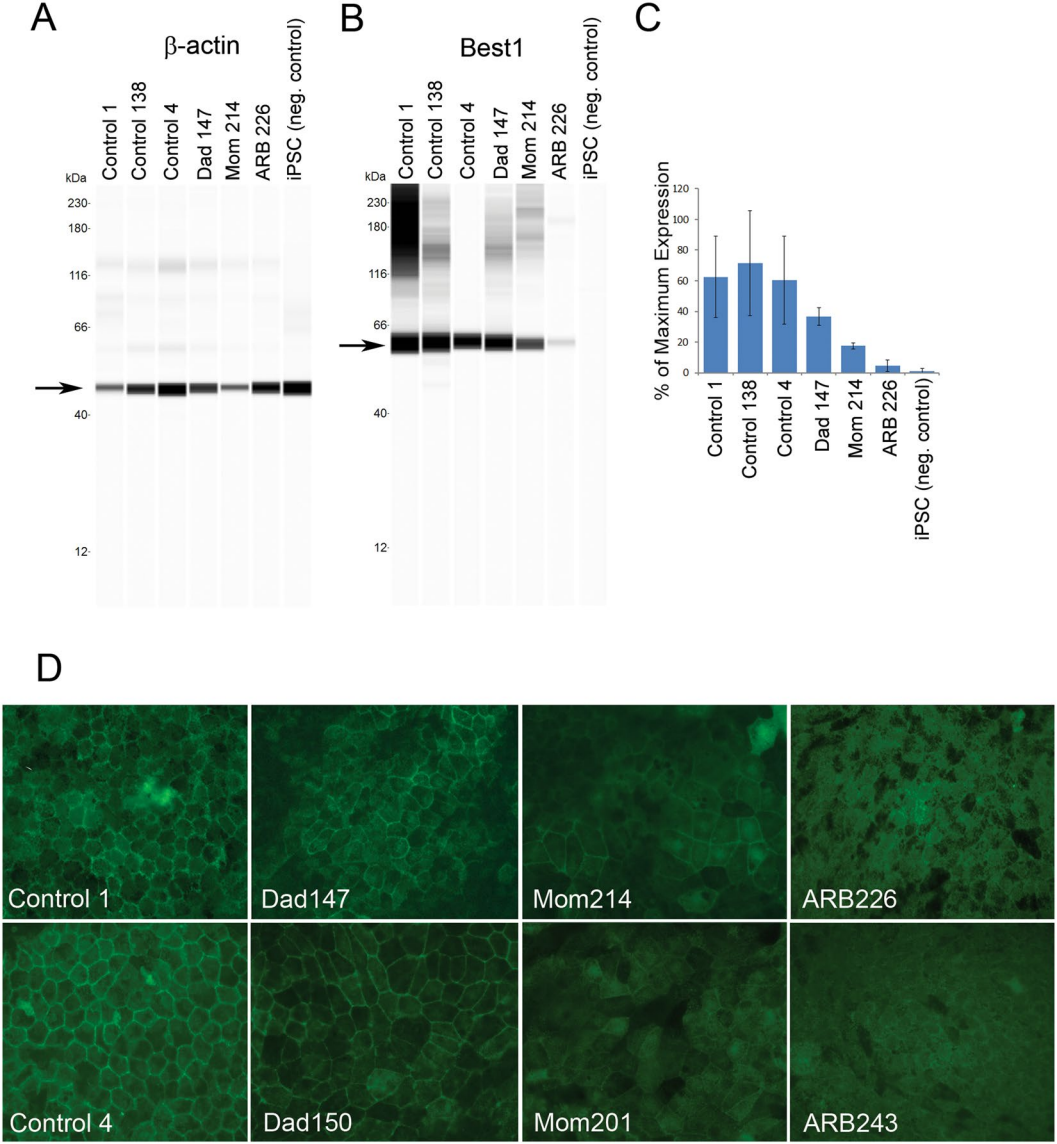
iPSC-RPE from an ARB donor show reduced levels of Best1 expression compared to five different iPSC-RPE control lines. (**A**) To compare Best1 protein expression levels between different iPSC-RPE lines, ß-actin was used as a loading control. (**B**) While iPSCs displayed no detectable Best1 protein expression, all shown iPSC-RPE lines – regardless of donor origin – displayed detectable Best1 protein expression. (**C**) The three control iPSC-RPE lines derived from unrelated, unaffected donors all exhibited similar levels of Best1 protein expression. The dad exhibited reduced levels compared to the unrelated controls and the mom showed levels that were about 50% that of the dad. This was expected as the antibody used cannot detect the mutant Best1^I366fsX18^ which both the mom and the ARB patient are heterozygous for. Best1 expression levels in the ARB iPSC-RPE line were about a quarter of the levels observed in the Mom 214 iPSC-RPE clone. (**D**) Widefield immunofluorescent staining of Best1 reveals basolateral staining in each of the iPSC-RPE lines, indicating that Best1 protein is properly localized regardless if Best1^R141H^ and/or Best1^I366fsX18^ are present. Immunofluorescent signal was notably weaker in the ARB iPSC-RPE clones than in the control clones.

Using ß-actin as a loading control (Fig. 5A), we compared protein expression levels of Best1 between the ARB patient, her parents, and three unrelated, unaffected controls. The ability to detect all Best1 in the subject and her mother was complicated by the fact that all available antibodies against human Best1 recognize regions of the C-terminal domain that are deleted from the Best1^I366fsX18^ mutant. Despite this, we were able to detect Best1 in lysates of Mom214 (Fig. 5B) and Mom201. Similarly, we detected Best1 protein in ARB226 (Fig. 5B) and ARB17 (Supplementary Figure S1). Best1 protein levels were extremely low and difficult to detect by Western blotting in ARB243 iPSC-RPE (Supplementary Figure S1). Best1 was expressed by each of the controls and also expressed in iPSC-RPE originating from the patient’s father (Fig. 5B). Interestingly, the level of Best1 expression was lower in the subject and her parents than in our unrelated, unaffected controls (Fig. 5C). iPSC-RPE cells from Mom214 appeared to express approximately ½ the amount of Best1 observed in Dad147, suggesting an even distribution of Best1 and Best1^I366fsX18^. The even lower level of Best1 expression in ARB clones compared to Mom214, however, is likely not explained by our inability to detect the Best1^I366fsX18^ mutant.

Using immunofluorescence staining, we next investigated the localization of Best1 and the Best1^R141H^ mutant. Best1 normally localizes to the basolateral plasma membrane of the RPE^14^. As shown in Fig. 5D, Control 1 and Control 4 iPSC-RPE exhibited immunofluorescent staining of Best1 at the basolateral plasma membrane (Fig. 5D). Dad147 and Dad150 showed a similar pattern of staining to Control 1 and Control 4, indicating that Best1^R141H^ is properly localized (Fig. 5D). A unique chimerism was observed in the Best1 immunofluorescent staining of both Mom214 and Mom201 (Fig. 5D). We suggest that this is due to the inability of the anti-Best1 antibodies to detect Best1^I366fsX18^ and therefore due to different levels of WT and mutant Best1 expression in different cells. In ARB226, staining for Best1 was faint but detectable and exhibited a similar chimeric pattern to Mom214 with clusters of brighter Best1 positive cells surrounded by groups of cells staining less strongly (Fig. 5D). When we detected Best1 in ARB243, it was weak but exhibited a similar pattern to ARB226 (Fig. 5D). We did not have sufficient cells from ARB17 to perform immunofluorescence staining. Based on these data we conclude that some amount of Best1^R141H^ is properly localized to the basolateral plasma membrane in our ARB iPSC-RPE lines.

### Phagocytosis is impaired in ARB

OCT images of the posterior pole of the eye in patients with ARB are characterized by serous retinal detachments in which shaggy elongated and uneven photoreceptor outer segments (OS) are observed^11,12^. As we have shown in our recent publication^39^ as well as in Fig. 1 of this manuscript, OCT imaging of the ARB patient reveals abnormally elongated photoreceptor OS as well as serous retinal detachments in both the left and right eyes (Fig. 1E–H). In patients with BVMD, OS can also exhibit a shaggy elongated appearance^48^ and defective phagocytosis has been observed in iPSC-RPE from donors with BVMD^20^.

**Figure 6:**
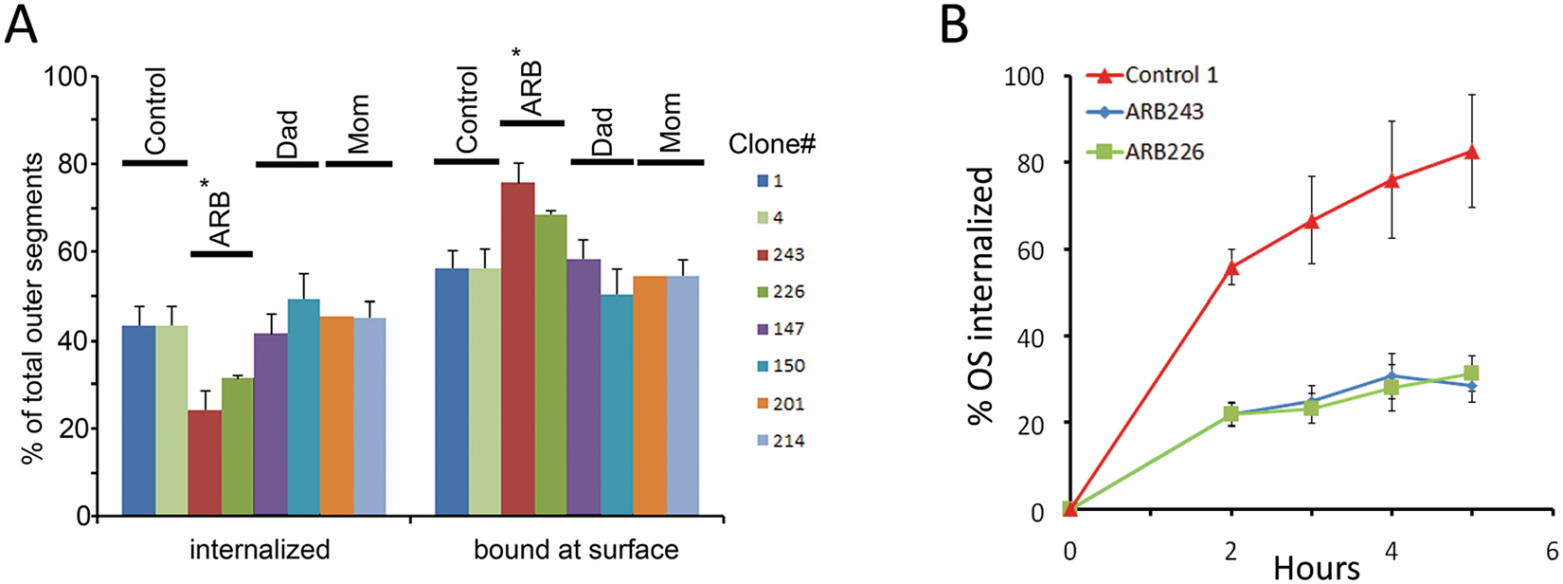
Phagocytosis of photoreceptor outer segments is impaired in ARB iPSC-RPE. Comparative phagocytosis assays using iPSC-RPE from our subject with ARB, her parents, and unrelated, unaffected controls were performed. This was done using a 96-well plate assay that provided two primary data points – the sum of fluorescence emissions of both bound and internalized OS. (**A**) We observed that, when challenged with FITC-labelled OS for three hours, approximately 42% of total OS were internalized in all control iPSC-RPE, regardless of the donor or the clone. In contrast, ARB243 exhibited only 24 ± 1% internalization and ARB226 exhibited only 31 ± 3% internalization (data are average ± SEM). Internalization of both ARB clones differed significantly from all other clones tested (p < 0.01). Since internalization of OS was significantly reduced, the percentage of OS that remained bound at the surface were significantly increased compared to the other iPSC-RPE lines (p < 0.015). (**B**) When examined in time course experiments, we observed that iPSC-RPE from the ARB subject differed substantially from controls. Control iPSC-RPE cells internalized > 75% of total OS within 5 hours while ARB iPSC-RPE internalized only ~40% of total OS within the same time frame. This was true for both ARB226 and ARB243.

To determine whether phagocytosis is impaired in ARB, we performed comparative phagocytosis assays using iPSC-RPE from our subject with ARB, her parents, and unrelated unaffected controls. To accomplish this we employed a 96-well plate based assay that provided three primary data points. These data points are the sum of the fluorescence emissions of bound and internalized OS as well as the fluorescence emissions of total internalized OS. We observed that, when challenged with FITC-labelled OS for three hours, approximately 42% of total OS were internalized in all control iPSC-RPE, regardless of the donor or the clone (Fig. 6A). In contrast, ARB243 exhibited only 24 ± 1% internalization and ARB226 exhibited only 31 ± 3% internalization (data are average ± SEM). Internalization of both ARB clones differed significantly from all other clones tested (p < 0.01) (Fig. 6A). Since internalization of OS was significantly reduced, the percentage of OS that remained bound at the surface were significantly increased compared to the other iPSC-RPE lines (p < 0.015) (Fig. 6A).

When examined in time course experiments (Fig. 6B), we observed that iPSC-RPE from the ARB subject differed substantially from controls. Control iPSC-RPE cells internalized >75% of total OS within 5 hours while ARB iPSC-RPE internalized only ~40% of total OS within the same time frame. This was true for both the ARB243 and ARB226 cell lines. For both Fig. 6A and B, the minor differences observed between ARB243 and ARB226 are likely due to clonal variation between different iPSC lines^49,50^.

## Discussion

ARB is a recessive disease due to mutations in the gene *BEST1*^4^. In this study we attempted to gain insight into the pathogenesis of ARB and better understand how its disease mechanisms are similar to or differ from the other bestrophinopathies. We did this by generating a “disease in a dish” model of ARB using iPSC-RPE from a pediatric subject with ARB due to compound heterozygous mutations in *BEST1*. These cells were compared to the patient’s parents as well as iPSC-RPE lines derived from three unrelated, unaffected individuals.

This was done first and foremost to test the hypothesis that ARB represents the human “null” phenotype and that, especially in compound heterozygotes harboring a truncating mutation, this occurs through NMD^4,33^. In our ARB patient compound heterozygous for the *BEST1* mutations R141H and I366fsX18 *BEST1*, mRNA for both R141H *BEST1* and I366fsX18 *BEST1* was easily detectable by both RT-PCR and qPCR. The levels of mRNA in the different ARB clones tested were comparable to those of the parents. While there was notable variability in mRNA levels between each of the unrelated, unaffected control lines, *BEST1* mRNA in ARB clone 226 was higher than mRNA in the normal iPSC-RPE lines Control 4 and Control 138 for Primer Set A. For Primer Set B, *BEST1* mRNA in ARB226 was higher than mRNA in Control 138. It is important to note that the variation observed in mRNA levels between the different controls is most likely normal iPSC variability due to genetic differences between individuals^49^. That *BEST1* mRNA was readily detectable in these ARB iPSC-RPE lines and, in some cases, notably higher than mRNA levels for other lines indicates that NMD is not occurring in our ARB iPSC-RPE model.

This is further emphasized by our finding that, just like the patient’s parents, Best1 protein is detectable via Western blotting in ARB iPSC-RPE. However, Best1 expression levels were higher in the control lines compared to iPSC-RPE generated from the subject as well as the subject’s parents. The Best1 expression levels in iPSC-RPE heterozygous for the I366fsX18 *BEST1* mutation were approximately half of the levels in iPSC-RPE heterozygous for the R141H *BEST1* mutation. Since the antibodies used only detect the C-terminus of WT Best1, this ~50% reduction is expected as the Best1^I366fsX18^ protein was undetectable in iPSC-RPE derived from the subject’s mother. We were surprised to observe that there was an approximate fourfold reduction in Best1 expression levels in the ARB subject’s iPSC-RPE compared to the mother’s iPSC-RPE. While the antibodies used could only detect Best1^R141H^ in ARB iPSC-RPE, we had expected to see a near-equivalent level of expression between iPSC-RPE derived from the ARB patient and the patient’s mother.

There are several possible explanations for why iPSC-RPE derived from this ARB patient showed reduced expression of Best1 compared to other iPSC-RPE lines. The first is that there is variability in expression levels between different iPSC lines, even between different clones derived from the same donor^49,50^. Exemplar of this, we were able to more readily detect Best1 protein in the ARB226 and ARB17 lines than in the ARB243 line. Another possibility is that the combination of Best1^R141H^ and Best1^I366fsX18^ leads to the formation of oligomers that are more prone to proteasomal degradation. We have shown that these two ARB mutants form hetero-oligomers with each other that are functionally active *in vitro*^39^, so it is likely that they are oligomerizing with each other *in vivo*. Moreover, others have identified specific ARB mutants that are subjected to proteasomal degradation when expressed in heterologous MDCK cells^38^. One of these mutants is Best1^R141H^. As such, it is possible that these mutant Best1^R141H^-Best1^I366fsX18^ oligomers are expressed but are more readily subjected to degradation due to misfolding or some other dysfunction. This increase in protein degradation would explain the reduced Best1 protein levels observed in ARB iPSC-RPE relative to iPSC-RPE from the subject’s parents as well as iPSC-RPE from unrelated controls.

More specifically, Uggenti *et al*. recently demonstrated that some of the mutations associated with ARB may be degraded by the ubiquitin proteasome system^38^. In that study they found that MDCK and HEK293 cells transfected to express mutant Best1 expressed more Best1 protein and had increased Best1-associated anion currents when treated with proteasome inhibitors. The concept of the ubiquitin/proteasome system degrading mutant Best1 could explain how mutations that do not ablate function might result, if not in a null phenotype, at least a reduction of Best1 protein below the threshold necessary for normal cellular function. Since our subject’s cells produced detectable Best1, but at levels significantly lower than either of her parents, it is possible that her cells do not produce enough Best1 protein to rise above a minimal threshold of Best1 activity necessary for normal cellular function. If this is the case, adding Best1 to the cells should rescue their functional deficit and halt vision loss. This could be accomplished by gene augmentation therapy, which is currently under investigation in dogs with cmr and shows promise in preventing loss of vision in those animals^9,51^. With the success of RPE65 gene therapy in the treatment of Leber’s congenital amaurosis^52,53^, it is easy to envision a gene therapy approach that could prevent vision loss in ARB. Alternatively, the amount of Best1 expressed could also be increased pharmacologically, perhaps with proteasome inhibitors as these were shown to be effective in transfected heterologous cells by Uggenti *et al*.^38^. If ARB is being caused by dysfunction induced by two recessive mutants working in concert, then gene therapy would clearly be the better choice as heterozygous parents of ARB patients have one copy of WT Best1 and show no symptoms of disease.

Though we have not yet had the opportunity to broadly characterize functional defects in ARB, we did observe that iPSC-RPE from the ARB subject exhibited impaired phagocytosis of photoreceptor OS. We identified that ARB iPSC-RPE show a significant reduction in the percentage of OS internalized after three hours. Time course experiments collecting multiple data points across five hours especially highlight this impairment in internalization. This is an exciting finding as Singh *et al*.^20^ have demonstrated delayed degradation of OS in iPSC-RPE from individuals with BVMD^20^. This indicates that impaired phagocytosis is implicated in the pathogenesis of both ARB and BVMD. We theorize that this defect in phagocytosis is due to either an insufficient amount of Best1 protein being available or due to dysfunction induced by the presence of two ARB mutants. For the latter, we know that both calcium and fluid transport play integral roles in RPE phagocytosis^12^. Since Best1 is both an anion channel and a regulator of intracellular calcium signaling, defects in these functions could indirectly lead to phagocytic defects. This is an important and novel finding which highlights that the relationship between ARB and BVMD needs further clarification. Though there are clear clinical differences (e.g., inheritance) between the diseases, the similarities observed in studies on iPSC-RPE suggest that the two diseases may share a more similar etiology than previously thought. This is further reinforced by a recent report by Gattoussi *et al*. which identified a family with BVMD due to the mutation F80I^54^. Several family members exhibited a fundus appearance that is indistinguishable from ARB^54^. Future studies are warranted to further investigate the pathogenic differences and similarities between these two diseases.

In summary, we set out to test the hypothesis that ARB is due to NMD of mutant *BEST1* mRNA leading to a null phenotype. We found that this hypothesis, in cells from an ARB patient carrying compound heterozygous mutations in *BEST1*, did not hold up. *BEST1* mRNA was abundant in the patient’s cells and Best1 protein was detected, albeit the latter at lower levels than in unrelated controls or in the subject’s parents. While further study is obviously necessary, we also identified a defect in the phagocytosis of photoreceptor OS that suggests a similar etiology to BVMD and which could potentially be exploited to test therapies for ARB *in vitro*. Based on our data, we conclude that ARB is neither due to NMD nor the complete absence of Best1 protein. However, the low level of Best1 protein found in these cells does suggest that increasing Best1 protein, either through gene augmentation therapy or by rescuing mutant Best1 from proteasomal degradation, may be a viable means of preventing vision loss in ARB.

## Materials and Methods

### Reprogramming and characterization of iPSCs

This study (NCT02162953, https://clinicaltrials.gov/ct2/show/NCT02162953) was performed in accordance with the Declaration of Helsinki and was approved by the Mayo Clinic Institutional Review Board, Mayo Clinic Stem Cell Research Oversight Committee, and the Mayo Clinic Center for Regenerative Medicine Biotrust Oversight group. All experimental methods were carried out in accordance with the approved guidelines. Briefly, dermal fibroblasts were isolated from 4 mm punch biopsies, expanded in DMEM (ThermoFisher Scientific, Waltham, Massachusetts, USA) with the addition of 10% FBS (ThermoFisher Scientific) and 1 × Antibiotic-Antimycotic containing amphotericin B, penicillin, and streptomycin (ThermoFisher Scientific). Fibroblasts were cryopreserved in Cell Recovery Freezing Medium (ThermoFisher Scientific). IPSCs were generated using a Sendai virus-based methodology (CytoTune-iPS 2.0, Invitrogen, Carlsbad, California, USA) by a third party vendor (ReGen Theranostics, Rochester, Minnesota, USA). Each line’s identity was confirmed by STR fingerprinting (Cell Line Genetics, Madison, Wisconsin, USA). IPSC clones were karyotyped (Mayo Clinic Medical Cytogenetics Core) and the expression of pluripotency markers Oct4, SSEA4, Nanog and Tra-1-60 was confirmed by flow cytometry and immunohistochemistry. Additionally, directed differentiation for each of the three germ layers was completed for each of the clones (Stemdiff, STEMCELL Technologies, Vancouver, British Columbia, Canada) as a measure of stemness. IPSC cultures were maintained on Geltrex coated plates in mTeSR1 (STEMCELL Technologies) at 37 °C in a 95% air/5% CO_2_ atmosphere and passaged using ReLeSR (STEMCELL Technologies).

### iPSC-RPE differentiation

Unrelated, unaffected control iPSC-RPE (Table 1) were differentiated by LAgen Laboratories (Rochester, Minnesota, USA). iPSCs from a patient with ARB and her parents were differentiated to RPE according to a modification of the method of Maruotti *et al*.^55^ as described by Johnson *et al*.^39^. In brief: Following 50 days in (DMEM/F12 or KO DMEM containing 15% (v/v) KO serum, 1% (v/v) nonessential amino acids, 1% (v/v) glutamine, 1% (v/v) antibiotic/antimycotic, and 0.1 mM 2-mercaptoethanol) iPSCs were differentiated to RPE using a basal differentiation media (RPEM) obtained from LAgen Laboratories (Rochester, Minnesota, USA) and supplemented with 2% (v/v) B27 (ThermoFisher Scientific) and 1% (v/v) antimycotic/antibiotic (ThermoFisher Scientific) for 30 days following plating. After 30 days, cells were maintained in RPEM supplemented with either 5% fetal bovine serum or 4% PLTMax® (LAgen Laboratories). For all experiments, differentiated RPE were grown for ≥two months.

### RT-PCR and qPCR

To isolate RNA and synthesize cDNA, iPSC-RPE monolayers were rinsed two times with 1 × DPBS plus calcium and magnesium. The cells were harvested by scraping them into 1 × DPBS without calcium and magnesium. Cells were centrifuged for 5 minutes at 5,000 × g at 4 °C. After aspirating the supernatant, cells were lysed in TRIzol® (Ambion, Carlsbad, California, USA). Total RNA was isolated using RNA Clean and Concentrator-5 kit (Zymo Research, Irvine, California, USA). The yield of RNA was determined using a Nanodrop spectrophotometer. Total RNA was treated with DNAse I using RNAse-free DNAse I according to the manufacturer’s protocol. CDNA was synthesized from two micrograms of oligo-dT primed total RNA using SuperScript III reverse transcriptase (ThermoFisher Scientific).

For our RPE genetic screen, primer pairs were designed using Primer-BLAST software^56^. At least one primer in the pair spanned an exon junction. Primer sequences directed towards the Sendai viral factors were from the CytoTune™-iPS 2.0 Sendai Reprogramming Kit (LifeTechnologies). All primers were ordered from Integrated DNA Technologies (Coralville, Iowa, USA). PCR reactions were batched according to the annealing temperature of the primer sets. Forty cycles of PCR using 10–100 ng of input cDNA and PCR was performed on an Applied Biosystems QuantStudio 5 qPCR instrument using PowerUp Sybr Green Master Mix (Applied Biosystems, Irvine, Texas, USA). A gene was deemed present if the C_T_ was less or equal to than 37 cycles.

For our qPCR experiments, reactions were performed in quadruplicate using primer pairs designed using Primer-BLAST software^56^. At least one primer in the pair spanned an exon junction. PCR was performed on an Applied Biosystems QuantStudio 5 qPCR instrument using PowerUp Sybr Green Master Mix (Applied Biosystems). 10 ng of cDNA was used as a template. The concentration of the primers was 0.5 µM and the annealing temperature was 55 °C.

### VEGF and PEDF secretion

Secretion of PEDF and VEGF were determined by ELISA using kits obtained from R&D Systems (Minneapolis, Minnesota, USA) and ThermoFisher Scientific, respectively.

### Measurement of TER

As we have done previously^39^, the TER of iPSC-RPE monolayers grown on Matrigel coated Transwell® (Corning, Tewksbury, MA, USA) supports was determined using a EVOM2 epithelial voltage/ohm meter with chopstick electrodes (World Precision Instruments, Sarasota, FL, USA). The resistance of a Transwell® with no cells was subtracted from the measured value and resistance was multiplied by the area in cm^2^ of the monolayer. Values shown in Table 3 are average ± SD for all monolayers of a given iPSC-RPE line and clone measured at eight weeks in culture.

### Western blotting

Western blots were performed using a WES SimpleWestern automated immunoblot system (ProteinSimple, San Jose, California, USA) according to the manufacturer’s instructions. In some instances, traditional Western blots were performed as described previously^39,57,58^. All antibodies used in this study are indicated in Supplementary Table 1.

### Immunofluorescence and transmitted light microscopy

Immunofluorescence was performed as described previously^39,57,58^. Immunofluorescence of Transwell filters (Corning, Tewksbury, Massachusetts, USA) was assessed 48 hrs after infection with specified adenoviral vectors. A rabbit, polyclonal antibody (ThermoFisher Scientific) was used to stain ZO-1 (1:100). Best1 was stained using either the rabbit, polyclonal antibody Pab125 (1:1000) or the mouse, monoclonal antibody E6-6 (1:1000), both of which have been previously described^14^. Widefield immunofluorescent as well as transmitted light images were obtained on an upright Nikon E600 microscope (Nikon Instruments, Melville, New York, USA).

### Phagocytosis

Assays of photoreceptor outer segment (OS) phagocytosis by iPSC-RPE were performed as described previously^39,59^. iPSC-RPE grown on 96-well plates were incubated with bovine OS labeled with FITC for either three hours or in time course experiments for 0, 1, 2, 3, or 5 hrs, after which they were washed with ice cold PBS 1 × containing 0.13 mM CaCl_2_ and 1 mM MgCl_2_ (PBS-CM). To determine the number of internalized vs. total OS, ½ of the wells at each time point were incubated for 10 mins with 0.2% trypan blue in PBS-CM to quench the fluorescence of OS that had not been internalized. Following a series of washes in PBS-CM, cells were fixed in 4% paraformaldehyde in PBS-CM and then washed in PBS-CM. Fluorescence emissions were quantified using a SpectraMax i3 plate reader and SoftMax Pro 6.4 software (SpectraMax, Molecular Devices, Sunnyvale, California, USA).

## Electronic supplementary material

Supplementary Data
