# Supplementary material for: Mutant Best1 Expression and Impaired Phagocytosis in an iPSC Model of Autosomal Recessive Bestrophinopathy
Source: Sci Rep. 2018 Mar 14;8:4487. doi: 10.1038/s41598-018-21651-z (PMC5852082; doi:10.1038/s41598-018-21651-z)
Supplement: Supplementary file 1 — Supplementary Data [file 41598_2018_21651_MOESM1_ESM.pdf]

SUPPLEMENTARY DATA

Mutant Best1 Expression and Impaired Phagocytosis in an iPSC Model of Autosomal Recessive Bestrophinopathy

[1*]Alan D. Marmorstein, PhD; [1,2]Adiv A. Johnson, PhD; [1]Lori A. Bachman; [1]Cynthia Andrews-Pfannkoch; [1]Travis Knudsen; [1]Benjamin Gilles; [1]Matthew S. Hill; [1]Jarel K. Gandhi, PhD; [1]Lihua Y. Marmorstein, PhD; [3,4,5]Jose S. Pulido, MD, MBA, MPH, MS

From the Departments of [1]Ophthalmology Research, [2]Molecular Medicine, [3]Ophthalmology, [4]Ocular Oncology, and [5]Vitreoretinal Diseases, Mayo Clinic, Rochester, MN

[*]Corresponding Author:

Alan D. Marmorstein, PhD
Mayo Clinic
Department of Ophthalmology Research
200 First Street, SW
Rochester, MN, 55905, USA
Tel: 507-284-2261
Fax: 507-284-5866
Email: Marmorstein.Alan@mayo.edu

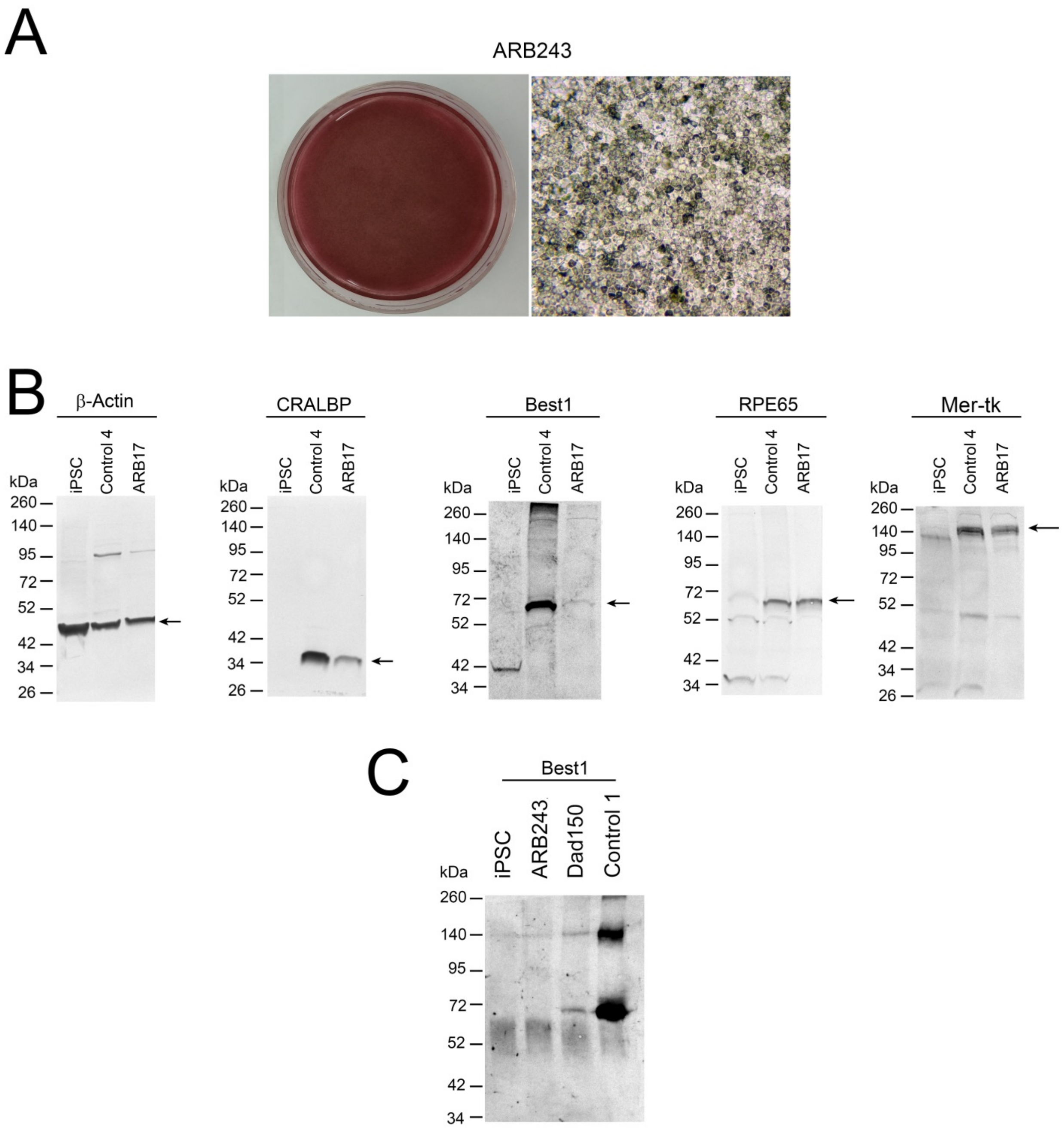

Supplementary Fig. 1: Expression of Best1 in other iPSC-RPE clones. A) The ARB iPSC-RPE clone ARB 243 showed dense pigmentation typical of cultured RPE. B) Just like control iPSC-RPE (positive control) but unlike iPSCs (negative control), ARB 17 iPSC-RPE showed expression of RPE65, CRALBP, Best1, and Mer-tk. ß-actin was used as a loading control for these comparative Western blots. C) Best1 expression was not readily detectable in the ARB 243 clone. Best1 expression was detectable in the Dad150 clone; thus, the protein levels that were observed were greatly reduced compared to those seen in Control 1 iPSC-RPE.

Supplementary Table 1: Antibodies used in this study.

Antigen	*Antibody*	*Host species*	*Clonality*	*Source*	*cat#*
Best1	E6-6	Mouse	Monoclonal	Marmorstein et al	na
Best1	Pab-125	Rabbit	Polyclonal	Marmorstein et al	na
Best1	na	Rabbit	Polyclonal	LAgen Laboratories (Rochester, MN)	016-Best1-01
β-Actin	AC15	Mouse	Monoclonal	Novus Biologicals (Littleton, CO)	NB600-501
CRALBP	B2	Mouse	Monoclonal	Novus Biologicals (Littleton, CO)	NB100-74392
RPE65	401.8B11.3D9	Mouse	Monoclonal	Novus Biologicals (Littleton, CO)	NB100-355
Mer-tk	Y323	Rabbit	Monoclonal	Abcam (Cambridge, MA)	ab52968
ZO-1	na	Rabbit	Polyclonal	Life Technologies	40-2300
Ezrin	na	Rabbit	Polyclonal	Cell Signaling Technology (Danvers, MA)	3145

Supplementary Fig. 2: Karyotyping, pluripotency marker expression, and directed differentiation to gem layers for newly generated iPSC lines used in this study.

Karyotype Analysis

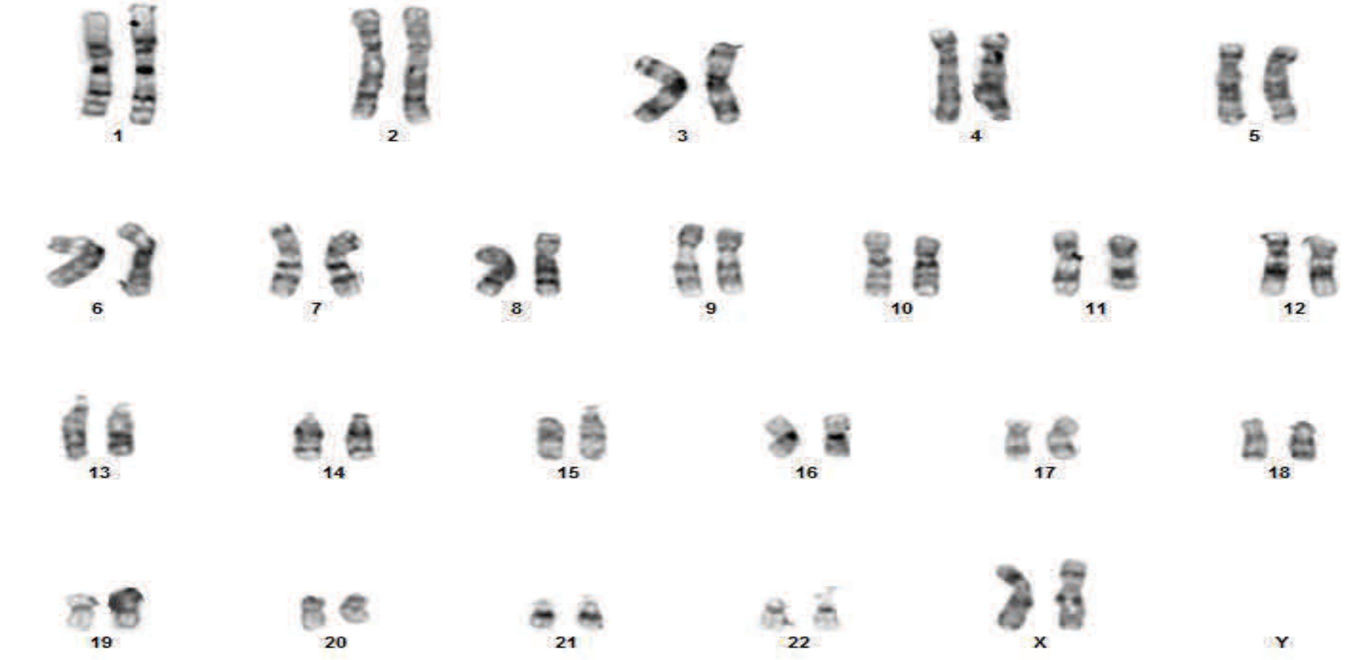

46,XX

MAYO CLINIC

Cytogenetics Laboratory

Mayo Clinic Laboratories-Rochester Main Campus, 200 First Street SW, Rochester, Minnesota 55905

Mayo Comp Cancer Center

**Name:** cl138p7 001-BIOTR-0001, ,	**DOB:** Not provided	**Collect Date:** 02/21/2014 12:00AM	**Requested By:** Dr. Zachary Resch
**Clinic #:**	**Age:**	**Rec'd Date:** 02/21/2014 3:58PM	**Location:** Rochester, MN
**Accession #:**	**Gender:** U	**Order Date:** 02/21/2014 3:58PM	**Source:** hiPSC
**Lab ID:** 956190	**Specimen:** Cultured cells		

REASON FOR REFERRAL

chromosome analysis

METHOD

Tumor culture

BANDING METHOD	CELLS ANALYZED	CELLS COUNTED	CELLS KARYOTYPED	EST. BAND RESOLUTION
GTL	20	0	2	
Total	20	0	2	400

RESULT

46,XX[20]

INTERPRETATION

NO CHARGE

No clonal abnormality was apparent.

Reviewed and approved by

Released: 03/03/2014 6:11PM

Patricia Greipp, DO

iPS Cell Pluripotency Marker Expression

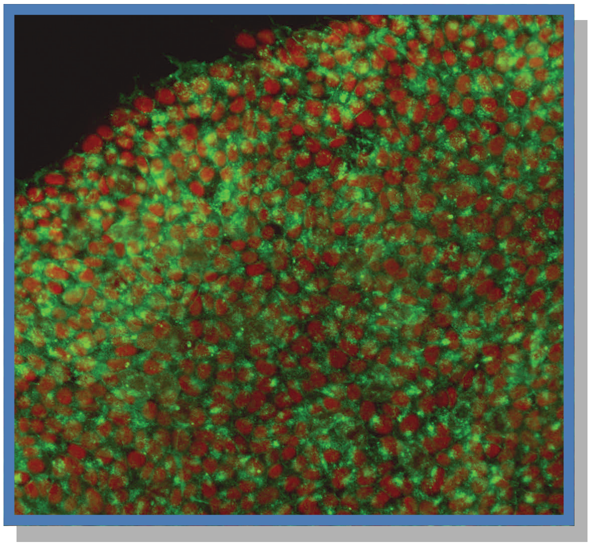

Clone-specific iPS cell colony (Passage ≤ 10) stained for the pluripotency markers Oct4 and SSEA with a nuclear counterstain (DAPI). 40X magnification.

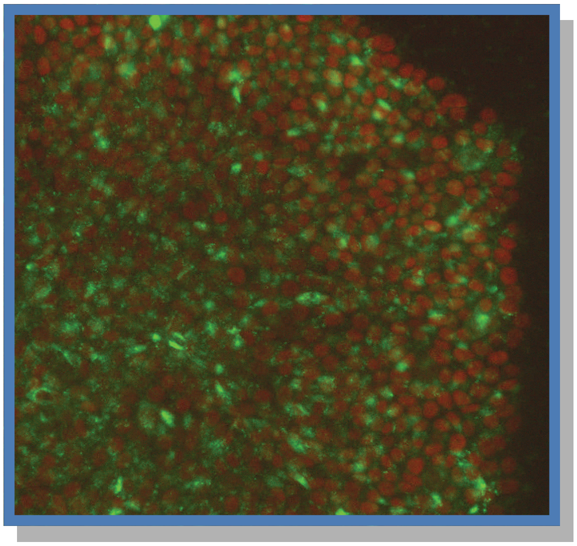

Clone-specific iPS cell colony (Passage ≤ 10) stained for the pluripotency markers Nanog and TRA-1-60 with a nuclear counterstain (DAPI). 20X magnification.

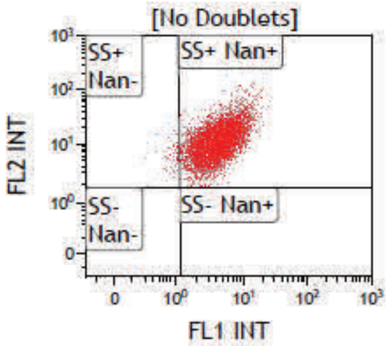

Gate	Number	%Gated
All	7,506	100.00
SS- Nan-	2	0.03
SS- Nan+	12	0.16
SS+ Nan-	95	1.27
SS+ Nan+	7,397	98.55

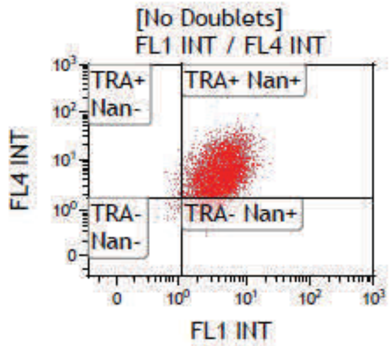

Gate	Number	%Gated
All	7,506	100.00
TRA- Nan-	54	0.72
TRA- Nan+	1,053	14.03
TRA+ Nan-	51	0.68
TRA+ Nan+	6,348	84.57

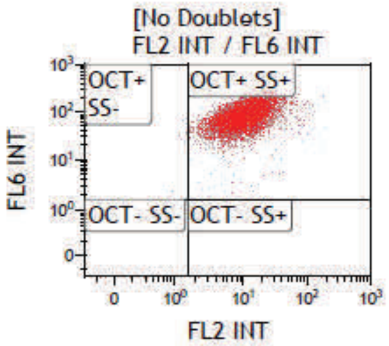

Gate	Number	%Gated
All	7,506	100.00
OCT- SS-	1	0.01
OCT- SS+	0	0.00
OCT+ SS-	11	0.15
OCT+ SS+	7,494	99.84

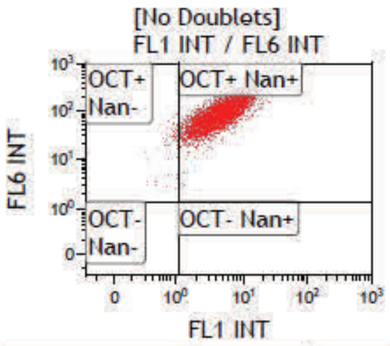

Gate	Number	%Gated
All	7,506	100.00
OCT- Nan-	1	0.01
OCT- Nan+	0	0.00
OCT+ Nan-	77	1.03
OCT+ Nan+	7,428	98.96

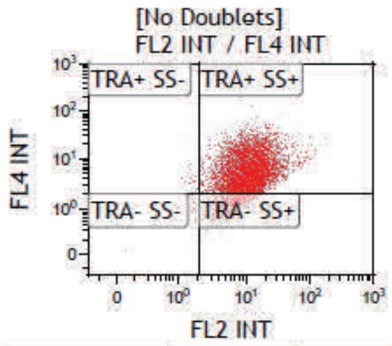

Gate	Number	%Gated
All	7,506	100.00
TRA- SS-	21	0.28
TRA- SS+	1,306	17.40
TRA+ SS-	24	0.32
TRA+ SS+	6,155	82.00

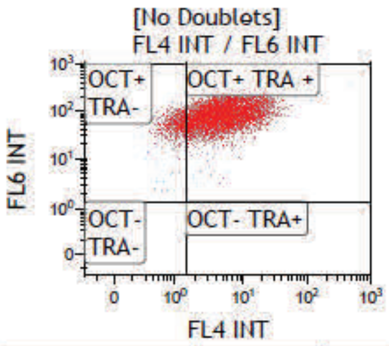

Gate	Number	%Gated
All	7,506	100.00
OCT- TRA-	1	0.01
OCT- TRA+	0	0.00
OCT+ TRA-	715	9.53
OCT+ TRA +	6,790	90.46

iPS Cell Directed Differentiation: Germ Layers

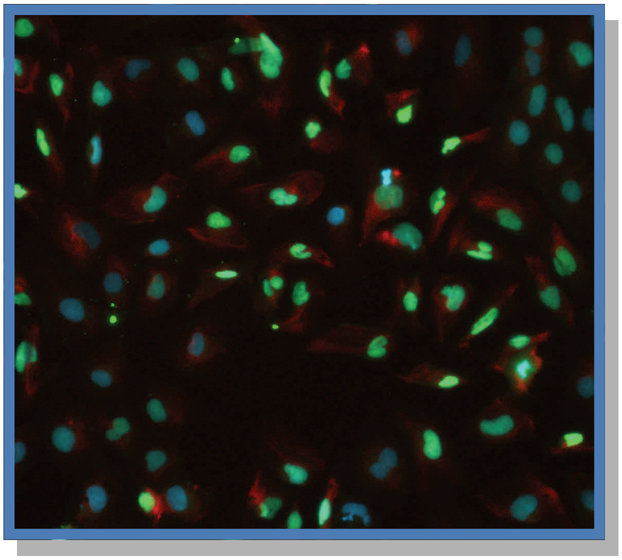

Ectoderm: *iPS cell clone underwent directed differentiation for 6 to 10 days, formalin fixed and Nestin and Pax-6 expression identified by immunohistochemistry. DAPI counterstain and image taken at 40X.*

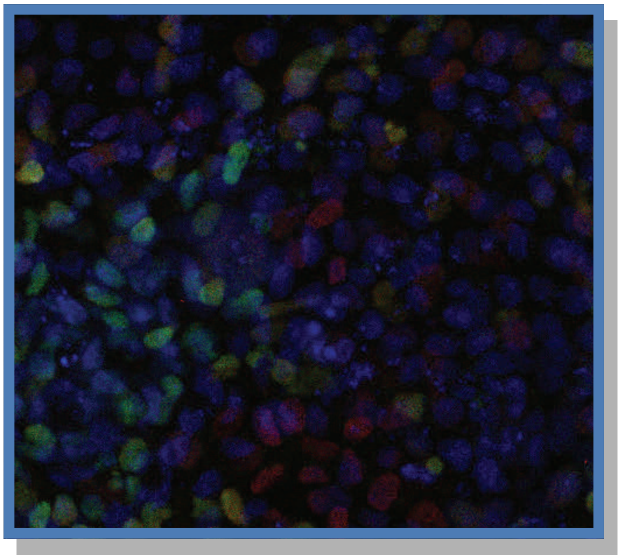

Endoderm: *iPS cell clone underwent directed differentiation for 5 days, formalin fixed and FoxA2 and SOX17 expression identified by immunohistochemistry. DAPI counterstain and image taken at 40X.*

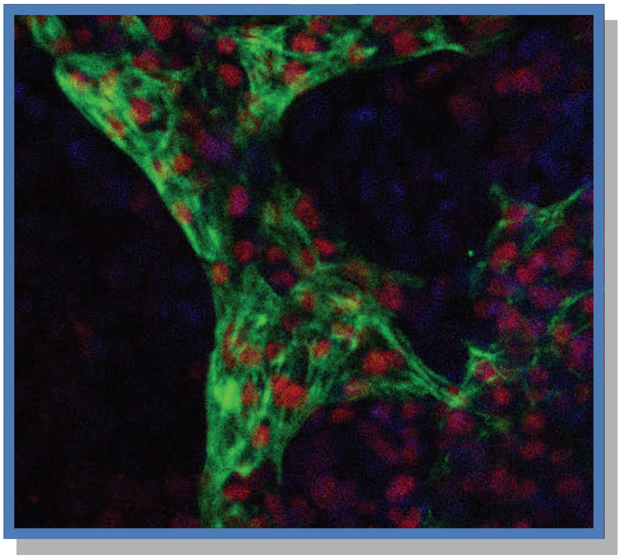

Mesooderm: *iPS cell clone underwent directed differentiation for 14 to 21 days, formalin fixed and NKX2.5 and TNNT expression identified by immunohistochemistry. DAPI counterstain and image taken at 40X.*

Karyotype Analysis

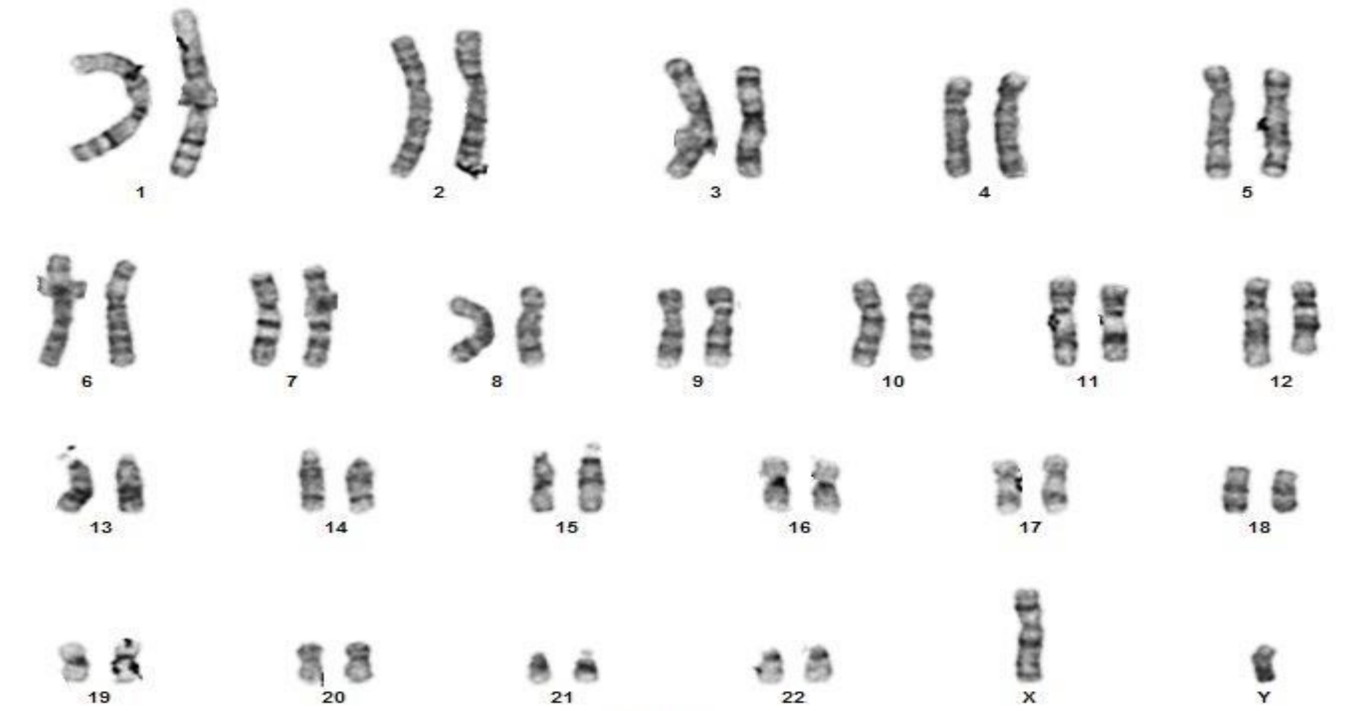

46,XY

MAYO CLINIC

Cytogenetics Laboratory

Mayo Clinic Laboratories-Rochester Main Campus, 200 First Street SW, Rochester, Minnesota 55905

Mayo Comp Cancer Center

Name: Cl. 147 p7 011-BIOTR-0001, , | **DOB:** Not provided | **Collect Date:** 10/01/2014 10:00AM | **Requested By:** Dr. Zachary Resch

Clinic #: | **Age:** | **Rec'd Date:** 10/01/2014 11:14AM | **Location:** Rochester, MN

Accession #: | **Gender:** U | **Order Date:** 10/01/2014 11:14AM | **Source:** hiPSC

Lab ID: 998779 | **Specimen:** Cultured cells

REASON FOR REFERRAL

chromosome analysis

METHOD

Tumor Culture

BANDING METHOD	CELLS ANALYZED	CELLS COUNTED	CELLS KARYOTYPED	EST. BAND RESOLUTION
GTL	20	0	2	
Total	20	0	2	400

RESULT

46,XY[20]

INTERPRETATION

NO CHANGE

No clonal abnormality was apparent.

Reviewed and approved by

Released: 10/27/2014 6:02PM

Patricia Greipp, DO

iPS Cell Pluripotency Marker Expression

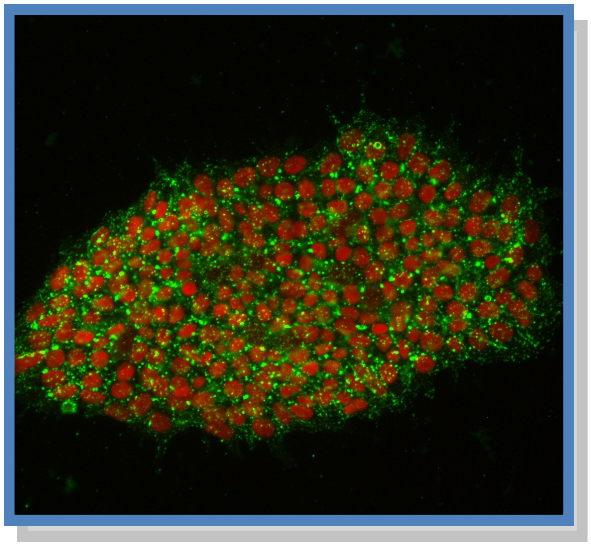

Clone-specific iPS cell colony (Passage ≤ 10) stained for the pluripotency markers Oct4 and SSEA with a nuclear counterstain (DAPI). 20X magnification.

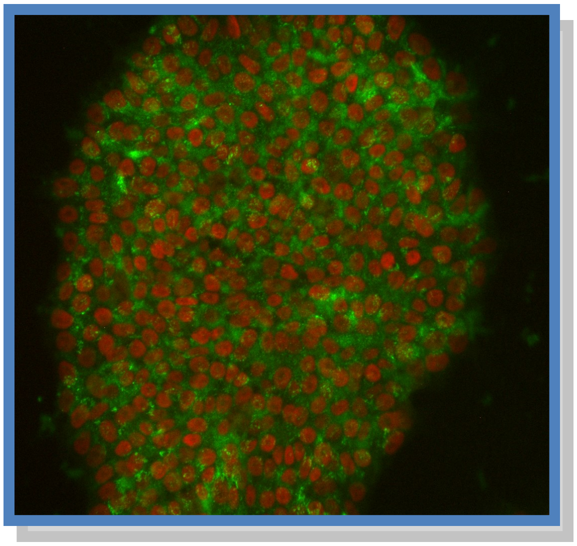

Clone-specific iPS cell colony (Passage ≤ 10) stained for the pluripotency markers Nanog and TRA-1-60 with a nuclear counterstain (DAPI). 20X magnification.

011-BIOTR-0001cl147 Stained 006
072313 4 Color 001 - Report Sheet 1

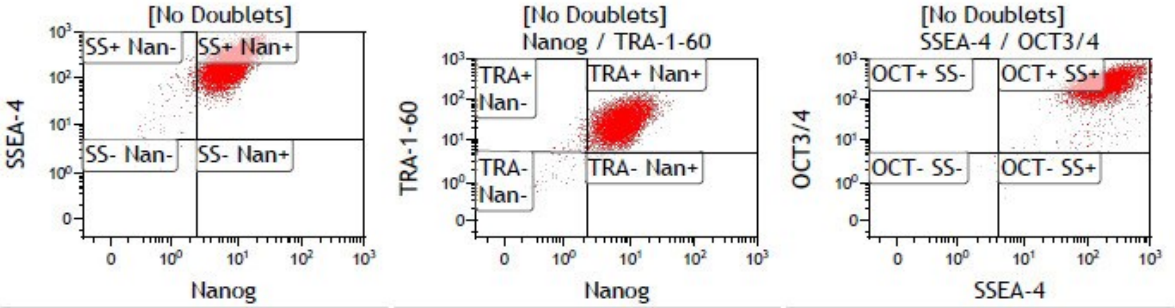

Gate	Number	%Gated
All	8,705	100.00
SS- Nan-	12	0.14
SS- Nan+	0	0.00
SS+ Nan-	175	2.01
SS+ Nan+	8,518	97.85

Gate	Number	%Gated
All	8,705	100.00
TRA- Nan-	57	0.65
TRA- Nan+	56	0.64
TRA+ Nan-	80	0.92
TRA+ Nan+	8,512	97.78

Gate	Number	%Gated
All	8,705	100.00
OCT- SS-	10	0.11
OCT- SS+	29	0.33
OCT+ SS-	0	0.00
OCT+ SS+	8,666	99.55

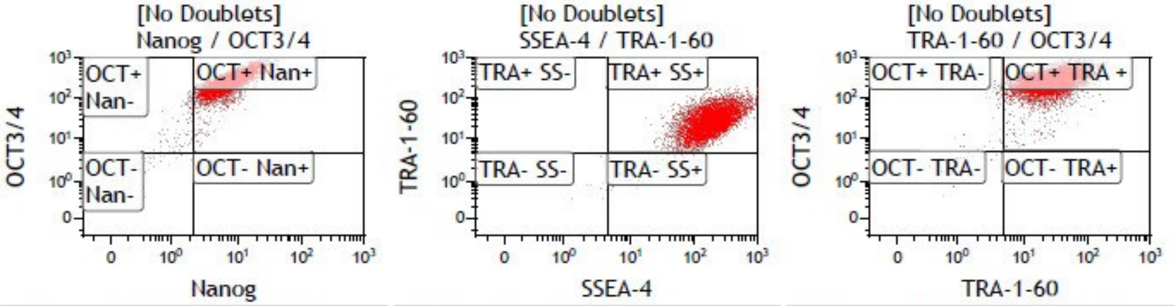

Gate	Number	%Gated
All	8,705	100.00
OCT- Nan-	36	0.41
OCT- Nan+	0	0.00
OCT+ Nan-	113	1.30
OCT+ Nan+	8,556	98.29

Gate	Number	%Gated
All	8,705	100.00
TRA- SS-	10	0.11
TRA- SS+	68	0.78
TRA+ SS-	0	0.00
TRA+ SS+	8,627	99.10

Gate	Number	%Gated
All	8,705	100.00
OCT- TRA-	35	0.40
OCT- TRA+	3	0.03
OCT+ TRA-	63	0.72
OCT+ TRA +	8,604	98.84

iPS Cell Directed Differentiation: Germ Layers

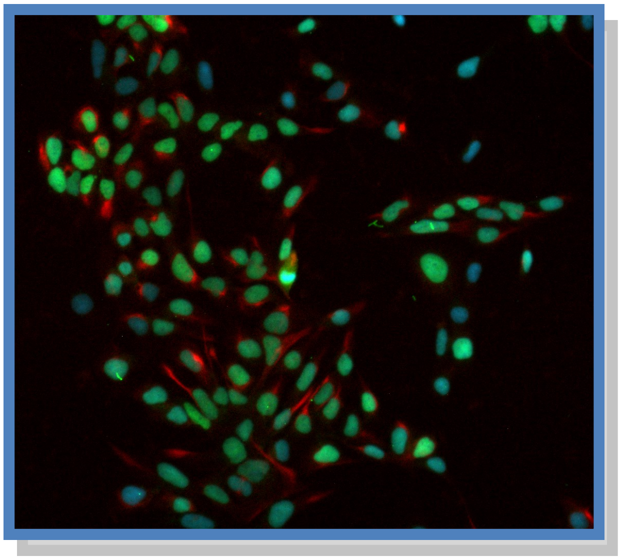

Ectoderm: *iPS cell clone underwent directed differentiation for 6 to 10 days, formalin fixed and Nestin and Pax-6 expression identified by immunohistochemistry. DAPI counterstain and image taken at 20X.*

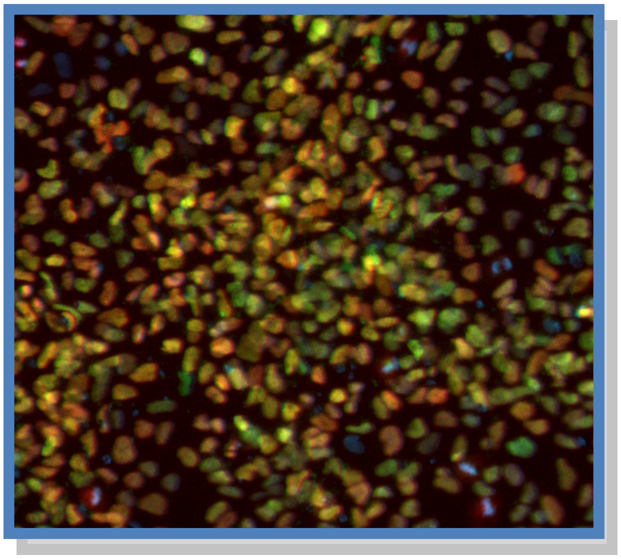

Endoderm: *iPS cell clone underwent directed differentiation for 5 days, formalin fixed and FoxA2 and SOX17 expression identified by immunohistochemistry. DAPI counterstain and image taken at 20X.*

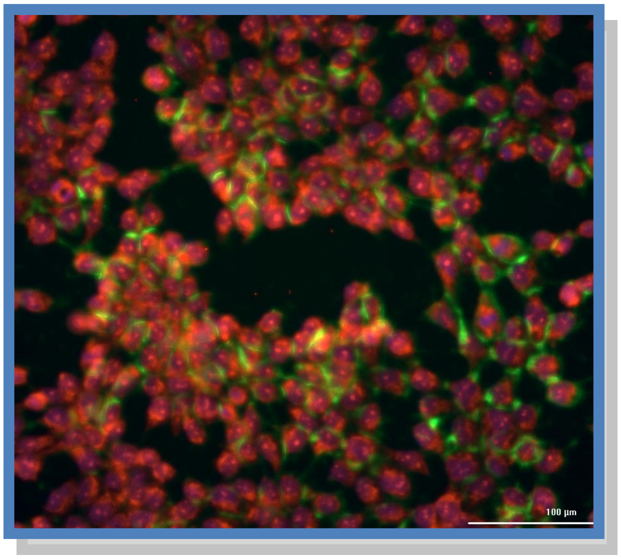

Mesooderm: *iPS cell clone underwent directed differentiation for 5 days, formalin fixed and CD31 and NCAM expression identified by immunohistochemistry. DAPI counterstain and image taken at 20X.*

Karyotype Analysis

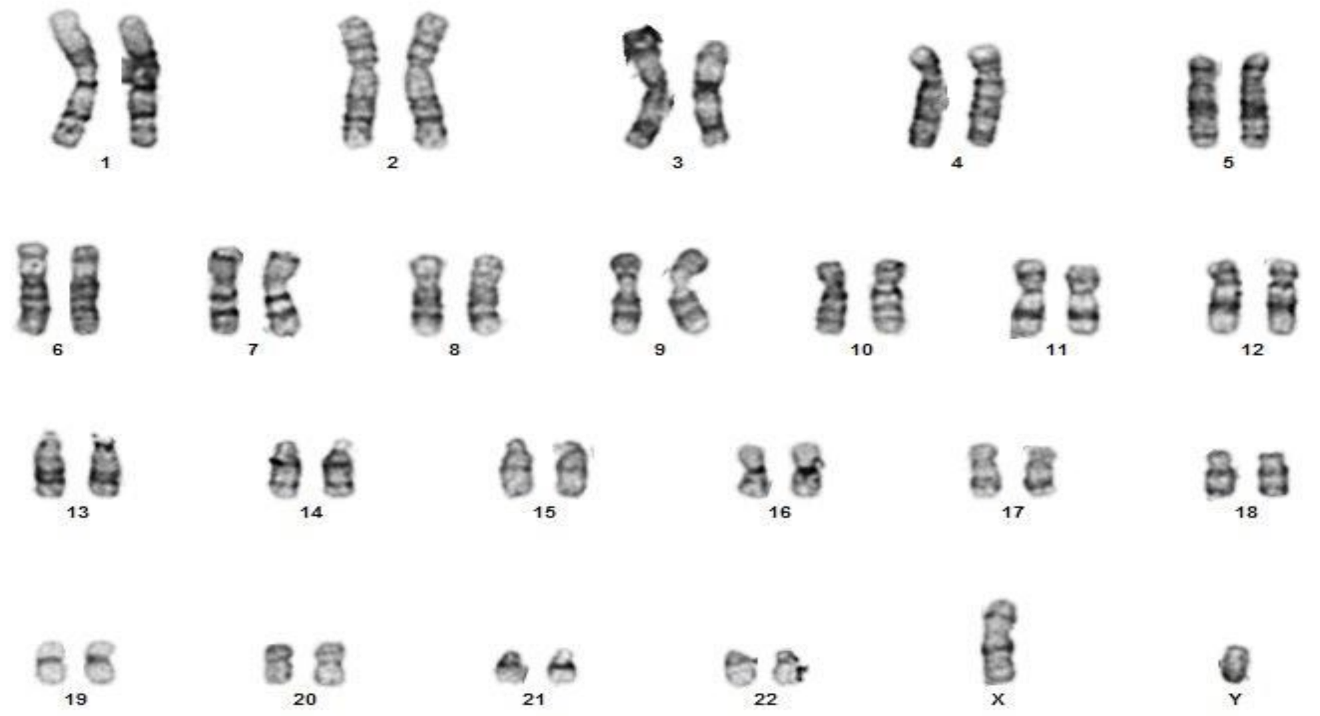

46,XY

MAYO CLINIC

Cytogenetics Laboratory
Mayo Clinic Laboratories-Rochester Main Campus, 200 First Street SW, Rochester, Minnesota 55905

Mayo Comp Cancer Center

**Name:** Cl. 150 p7 011-BIOTR-0001, ,	**DOB:** Not provided	**Collect Date:** 10/01/2014 10:00AM	**Requested By:** Dr. Zachary Resch
**Clinic #:**	**Age:**	**Rec'd Date:** 10/01/2014 11:18AM	**Location:** Rochester, MN
**Accession #:**	**Gender:** U	**Order Date:** 10/01/2014 11:17AM	**Source:** hiPSC
**Lab ID:** 998780	**Specimen:** Cultured cells		

REASON FOR REFERRAL

chromosome analysis

METHOD

Tumor culture

BANDING METHOD	CELLS ANALYZED	CELLS COUNTED	CELLS KARYOTYPED	EST. BAND RESOLUTION
GTL	20	0	2	
Total	20	0	2	400

RESULT

46,XY[20]

INTERPRETATION

NO CHANGE

No clonal abnormality was apparent.

Reviewed and approved by

Released: 10/27/2014 6:02PM

Patricia Greipp, DO

iPS Cell Pluripotency Marker Expression

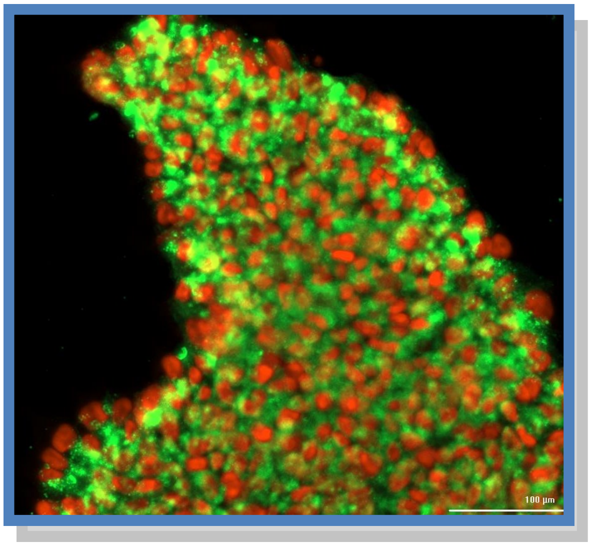

Clone-specific iPS cell colony (Passage ≤ 10) stained for the pluripotency markers Oct4 and SSEA with a nuclear counterstain (DAPI). 20X magnification.

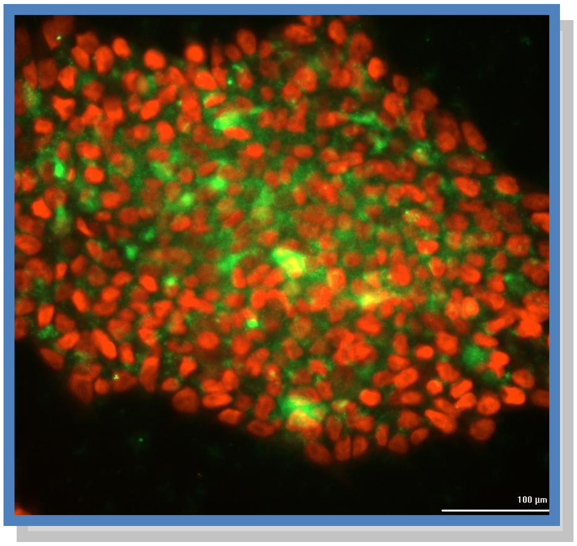

Clone-specific iPS cell colony (Passage ≤ 10) stained for the pluripotency markers Nanog and TRA-1-60 with a nuclear counterstain (DAPI). 20X magnification.

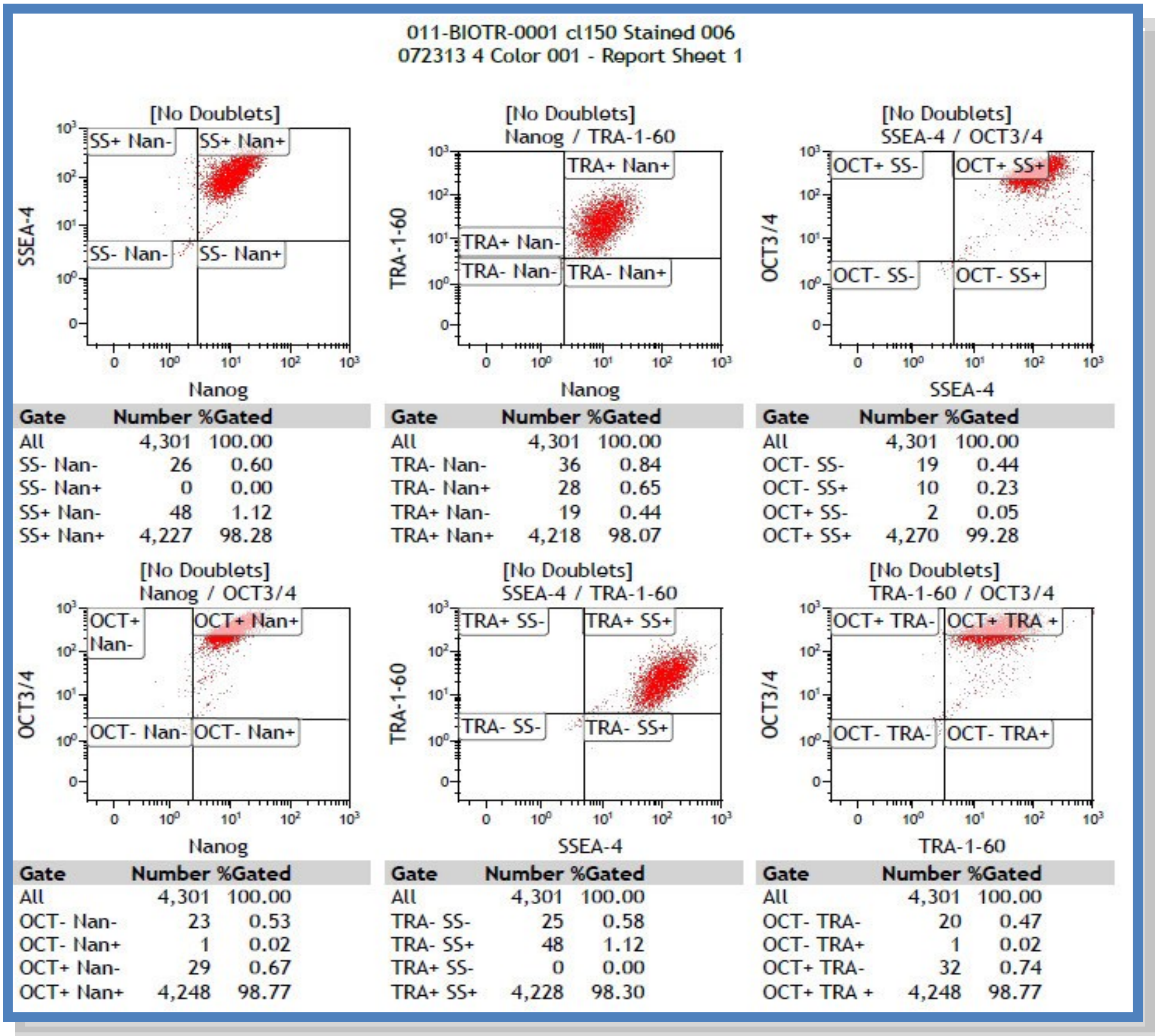
011-BIOTR-0001 cl150 Stained 006
072313 4 Color 001 - Report Sheet 1

[No Doublets] — SSEA-4 vs Nanog

Gate	Number	%Gated
All	4,301	100.00
SS- Nan-	26	0.60
SS- Nan+	0	0.00
SS+ Nan-	48	1.12
SS+ Nan+	4,227	98.28

[No Doublets] Nanog / TRA-1-60

Gate	Number	%Gated
All	4,301	100.00
TRA- Nan-	36	0.84
TRA- Nan+	28	0.65
TRA+ Nan-	19	0.44
TRA+ Nan+	4,218	98.07

[No Doublets] SSEA-4 / OCT3/4

Gate	Number	%Gated
All	4,301	100.00
OCT- SS-	19	0.44
OCT- SS+	10	0.23
OCT+ SS-	2	0.05
OCT+ SS+	4,270	99.28

[No Doublets] Nanog / OCT3/4

Gate	Number	%Gated
All	4,301	100.00
OCT- Nan-	23	0.53
OCT- Nan+	1	0.02
OCT+ Nan-	29	0.67
OCT+ Nan+	4,248	98.77

[No Doublets] SSEA-4 / TRA-1-60

Gate	Number	%Gated
All	4,301	100.00
TRA- SS-	25	0.58
TRA- SS+	48	1.12
TRA+ SS-	0	0.00
TRA+ SS+	4,228	98.30

[No Doublets] TRA-1-60 / OCT3/4

Gate	Number	%Gated
All	4,301	100.00
OCT- TRA-	20	0.47
OCT- TRA+	1	0.02
OCT+ TRA-	32	0.74
OCT+ TRA +	4,248	98.77

iPS Cell Directed Differentiation: Germ Layers

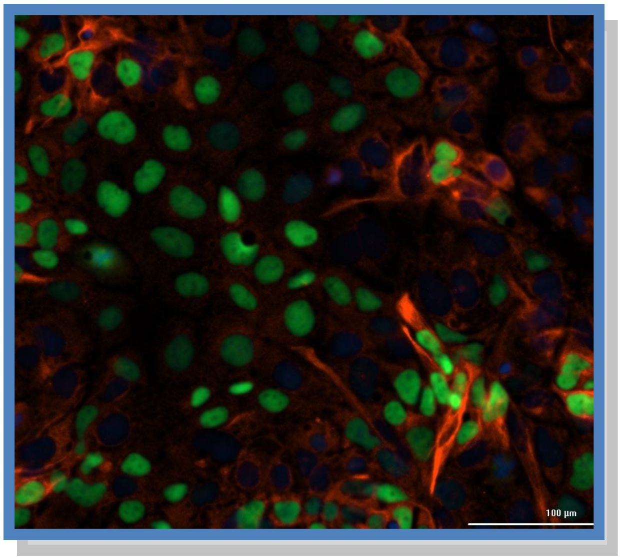

Ectoderm: *iPS cell clone underwent directed differentiation for 6 to 10 days, formalin fixed and Nestin and Pax-6 expression identified by immunohistochemistry. DAPI counterstain and image taken at 20X.*

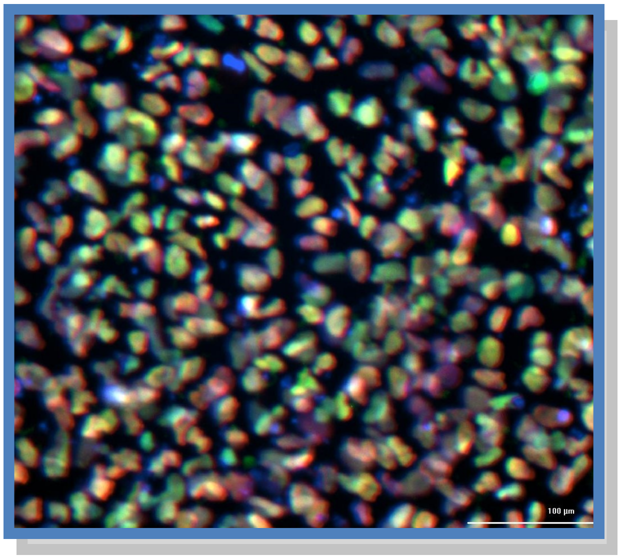

Endoderm: *iPS cell clone underwent directed differentiation for 5 days, formalin fixed and FoxA2 and SOX17 expression identified by immunohistochemistry. DAPI counterstain and image taken at 20X.*

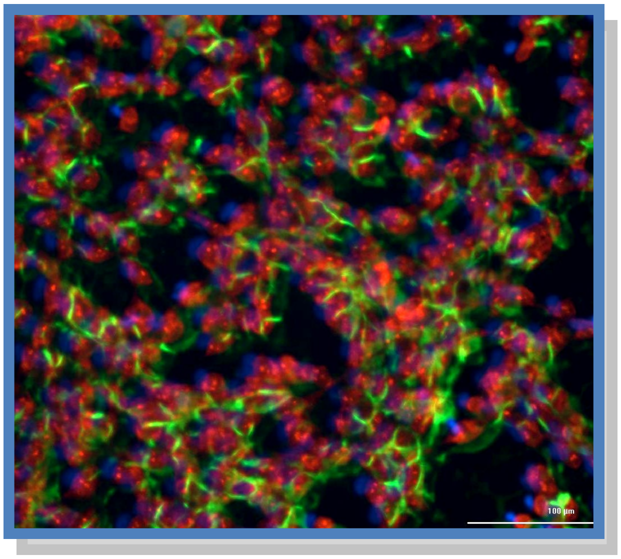

Mesooderm: *iPS cell clone underwent directed differentiation for 5 days, formalin fixed and CD31 and NCAM expression identified by immunohistochemistry. DAPI counterstain and image taken at 20X.*

Karyotype Analysis

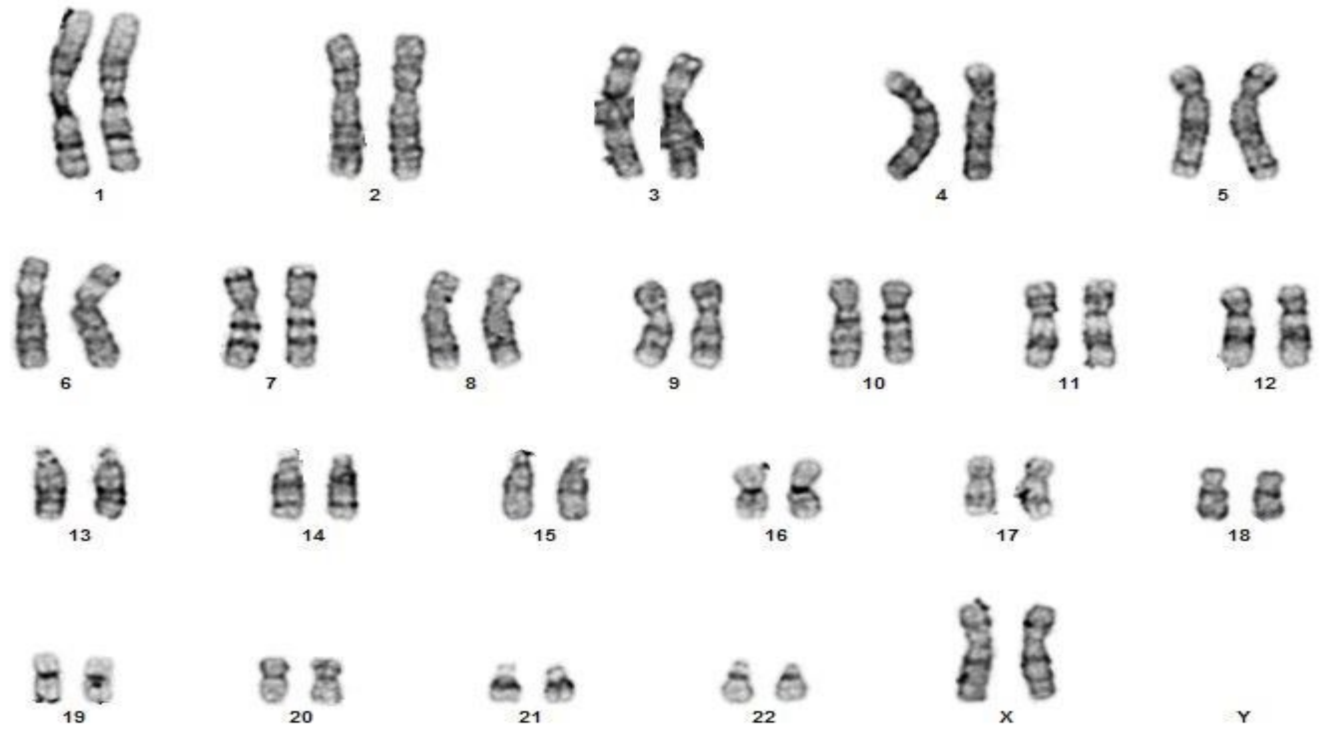

46,XX

MAYO CLINIC

Cytogenetics Laboratory

Mayo Clinic Laboratories-Rochester Main Campus, 200 First Street SW, Rochester, Minnesota 55905

Mayo Comp Cancer Center

**Name:** Cl. 201 p8 011-BIOTR-0002, ,	**DOB:** Not provided	**Collect Date:** 09/29/2014 12:00PM	**Requested By:** Dr. Zachary Resch
**Clinic #:**	**Age:**	**Rec'd Date:** 09/29/2014 12:23PM	**Location:** Rochester, MN
**Accession #:**	**Gender:** U	**Order Date:** 09/29/2014 12:22PM	**Source:** hiPSC
**Lab ID:** 998367	**Specimen:** Cultured cells		

REASON FOR REFERRAL

chromosome analysis

METHOD

Tumor culture

BANDING METHOD	CELLS ANALYZED	CELLS COUNTED	CELLS KARYOTYPED	EST. BAND RESOLUTION
GTL	20	0	2	
Total	20	0	2	400

RESULT

46,XX[20]

INTERPRETATION

NO CHARGE

No clonal abnormality was apparent.

Reviewed and approved by

Released: 11/10/2014 12:06PM

Patricia Greipp, DO

iPS Cell Pluripotency Marker Expression

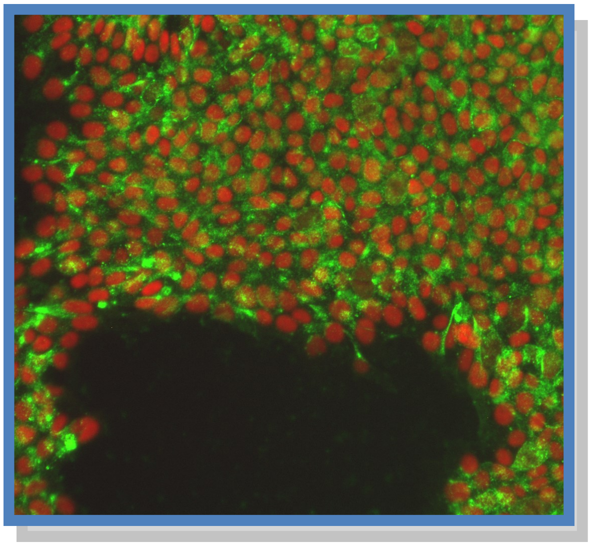

Clone-specific iPS cell colony (Passage ≤ 10) stained for the pluripotency markers Oct4 and SSEA with a nuclear counterstain (DAPI). 20X magnification.

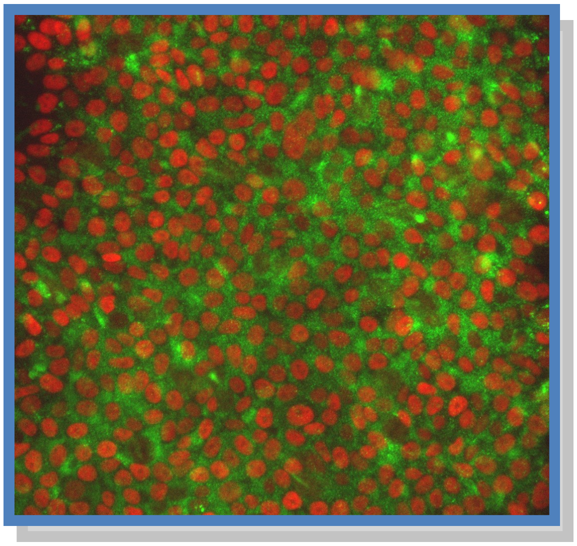

Clone-specific iPS cell colony (Passage ≤ 10) stained for the pluripotency markers Nanog and TRA-1-60 with a nuclear counterstain (DAPI). 20X magnification.

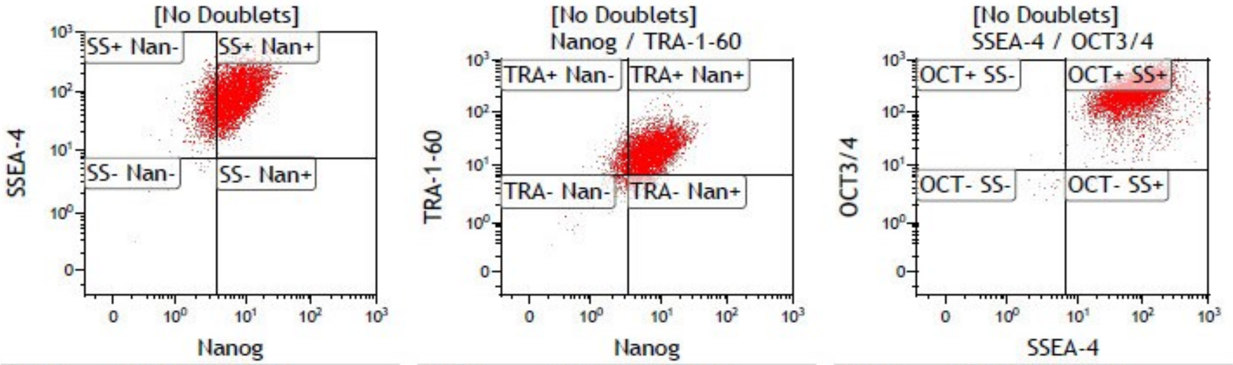

Gate	Number	%Gated
All	7,498	100.00
SS- Nan-	23	0.31
SS- Nan+	1	0.01
SS+ Nan-	1,249	16.66
SS+ Nan+	6,225	83.02

Gate	Number	%Gated
All	7,498	100.00
TRA- Nan-	213	2.84
TRA- Nan+	418	5.57
TRA+ Nan-	587	7.83
TRA+ Nan+	6,280	83.76

Gate	Number	%Gated
All	7,498	100.00
OCT- SS-	19	0.25
OCT- SS+	9	0.12
OCT+ SS-	2	0.03
OCT+ SS+	7,468	99.60

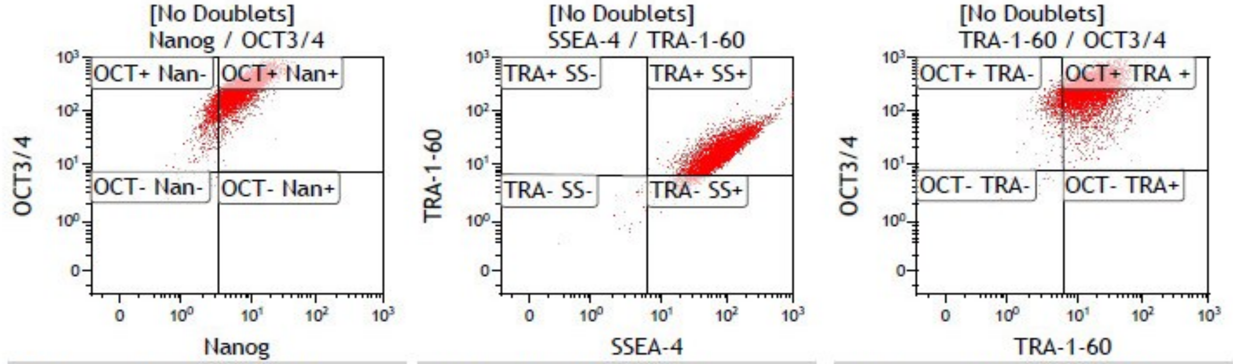

Gate	Number	%Gated
All	7,498	100.00
OCT- Nan-	25	0.33
OCT- Nan+	0	0.00
OCT+ Nan-	827	11.03
OCT+ Nan+	6,646	88.64

Gate	Number	%Gated
All	7,498	100.00
TRA- SS-	20	0.27
TRA- SS+	538	7.18
TRA+ SS-	0	0.00
TRA+ SS+	6,940	92.56

Gate	Number	%Gated
All	7,498	100.00
OCT- TRA-	23	0.31
OCT- TRA+	4	0.05
OCT+ TRA-	491	6.55
OCT+ TRA +	6,980	93.09

iPS Cell Directed Differentiation: Germ Layers

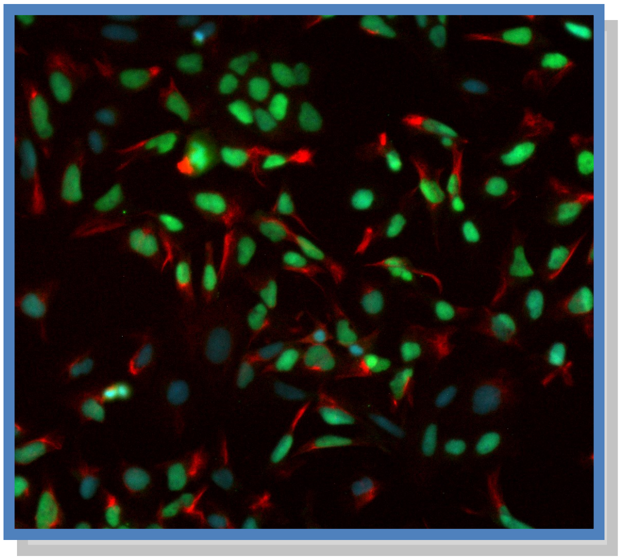

Ectoderm: *iPS cell clone underwent directed differentiation for 6 to 10 days, formalin fixed and Nestin and Pax-6 expression identified by immunohistochemistry. DAPI counterstain and image taken at 20X.*

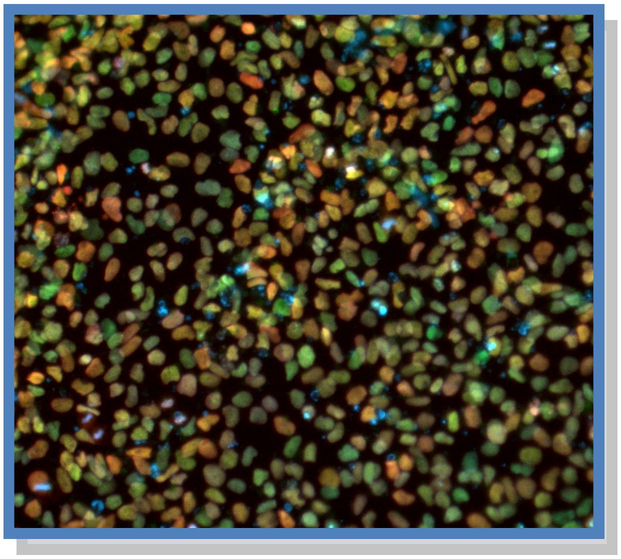

Endoderm: *iPS cell clone underwent directed differentiation for 5 days, formalin fixed and FoxA2 and SOX17 expression identified by immunohistochemistry. DAPI counterstain and image taken at 20X.*

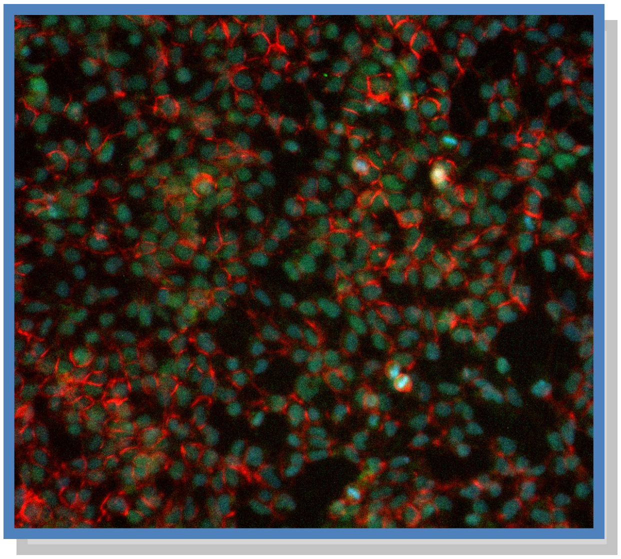

Mesooderm: *iPS cell clone underwent directed differentiation for 5 days, formalin fixed and NCAM and Brachyury expression identified by immunohistochemistry. DAPI counterstain and image taken at 20X.*

Karyotype Analysis

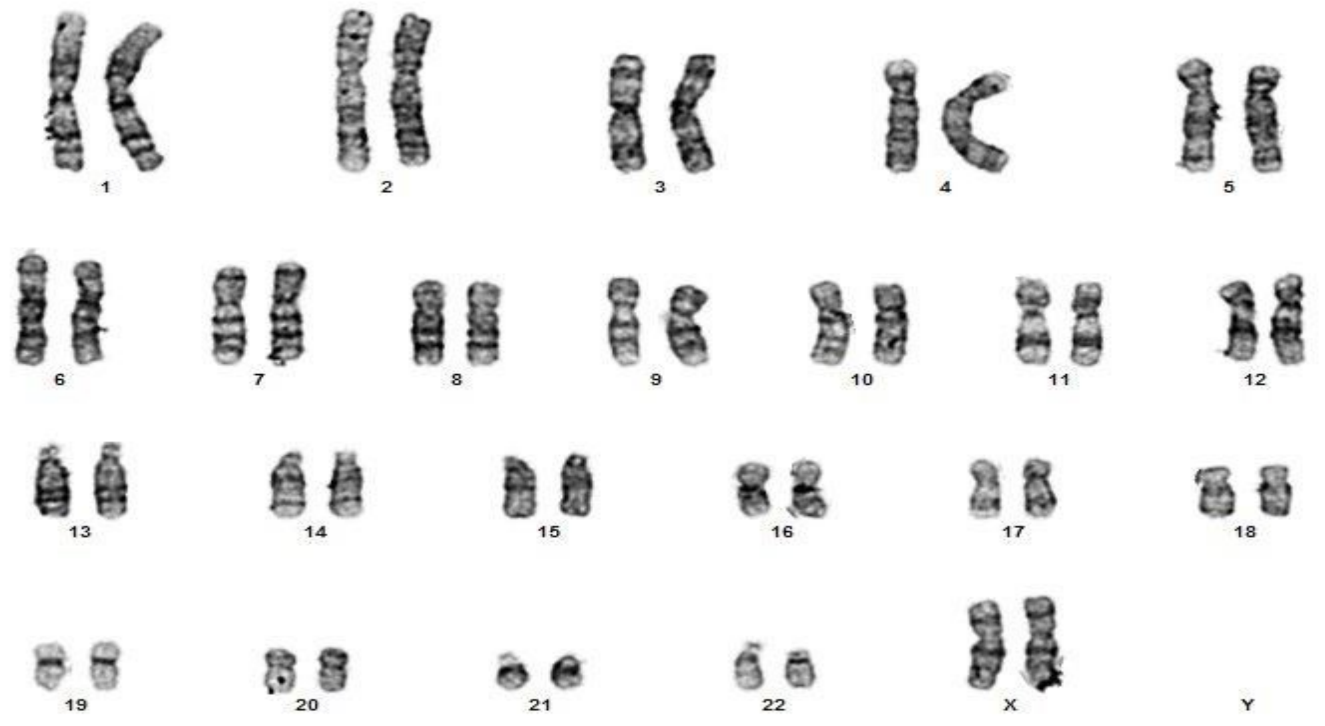

MAYO CLINIC

Cytogenetics Laboratory

Mayo Clinic Laboratories-Rochester Main Campus, 200 First Street SW, Rochester, Minnesota 55905

Mayo Comp Cancer Center

AMENDED REPORT

**Name:** 011-BIOTR-0002 Cl. 214, ,	**DOB:** Not provided	**Collect Date:** 09/25/2014 10:00AM	**Requested By:** Dr. Zachary Resch
**Clinic #:**	**Age:**	**Rec'd Date:** 09/25/2014 2:20PM	**Location:** Rochester, MN
**Accession #:**	**Gender:** U	**Order Date:** 09/25/2014 2:20PM	**Source:** hiPSC
**Lab ID:** 997700	**Specimen:** Cultured cells		

REASON FOR REFERRAL

chromosome analysis

METHOD

Tumor culture

BANDING METHOD	CELLS ANALYSED	CELLS COUNTED	CELLS KARYOTYPED	EST. BAND RESOLUTION
GTL	20	0	2	
Total	20	0	2	400

RESULT

46,XX[20]

INTERPRETATION

NO CHARGE

No clonal abnormality was apparent.

AMENDMENT

REPORT REVISED 08/03/2015: The 11/04/2014 report was revised to change the patient's name from Cl. 214 p7 011-BIOTR-0004 to 011-BIOTR-0002 Cl. 214. The result and interpretation remain unchanged.

Reviewed and approved by   Released: 08/05/2015 4:29PM

Patricia Greipp, DO

iPS Cell Pluripotency Marker Expression

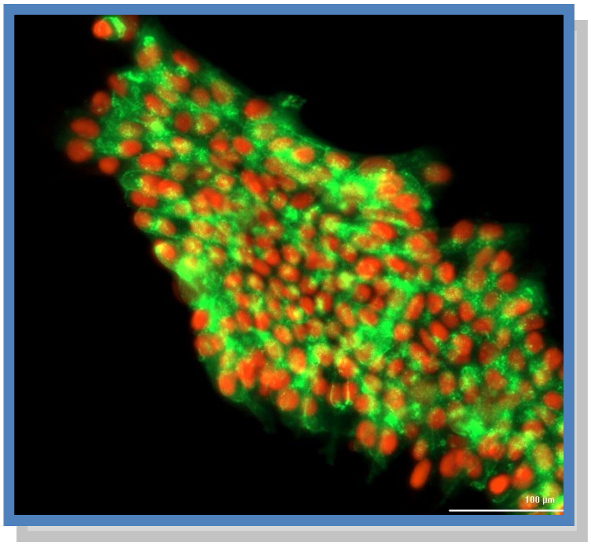

Clone-specific iPS cell colony (Passage < 10) stained for the pluripotency markers Oct4 and SSEA with a nuclear counterstain (DAPI). 20X magnification.

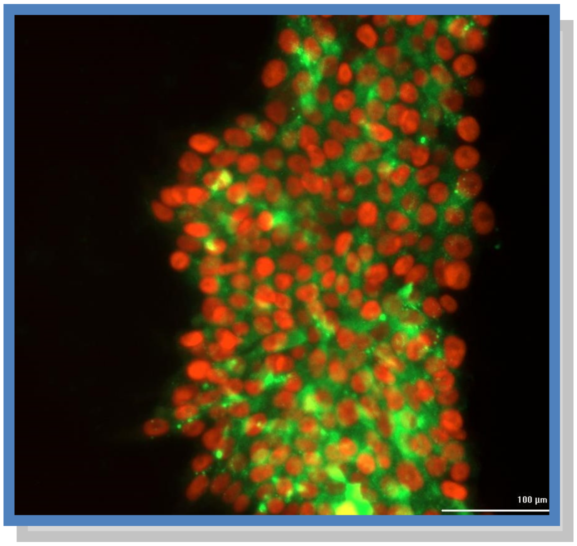

Clone-specific iPS cell colony (Passage < 10) stained for the pluripotency markers Nanog and TRA-1-60 with a nuclear counterstain (DAPI). 20X magnification.

011-BIOTR-0002 cl 214 p8 ReLe-pluri-2017-02-07-155557-2
072313 4 Color 001 - Report Sheet 1

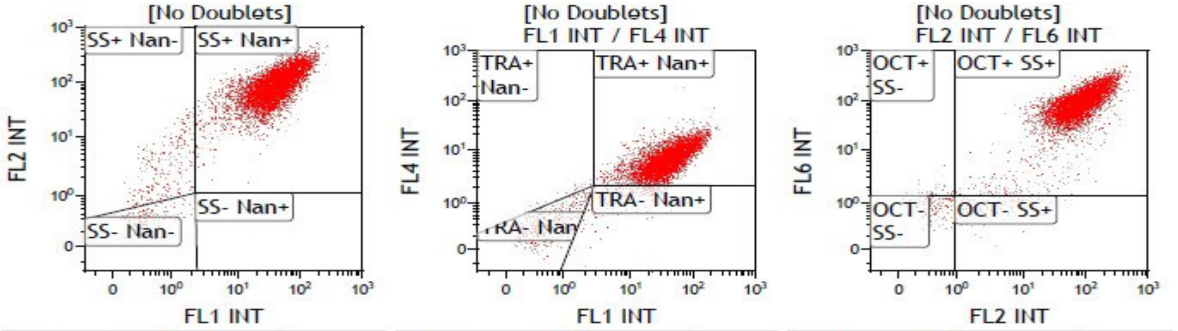

Gate	Number	%Gated
All	8,580	100.00
SS- Nan-	136	1.59
SS- Nan+	4	0.05
SS+ Nan-	264	3.08
SS+ Nan+	8,176	95.29

Gate	Number	%Gated
All	8,580	100.00
TRA- Nan-	369	4.30
TRA- Nan+	283	3.30
TRA+ Nan-	21	0.24
TRA+ Nan+	7,907	92.16

Gate	Number	%Gated
All	8,580	100.00
OCT- SS-	137	1.60
OCT- SS+	139	1.62
OCT+ SS-	33	0.38
OCT+ SS+	8,271	96.40

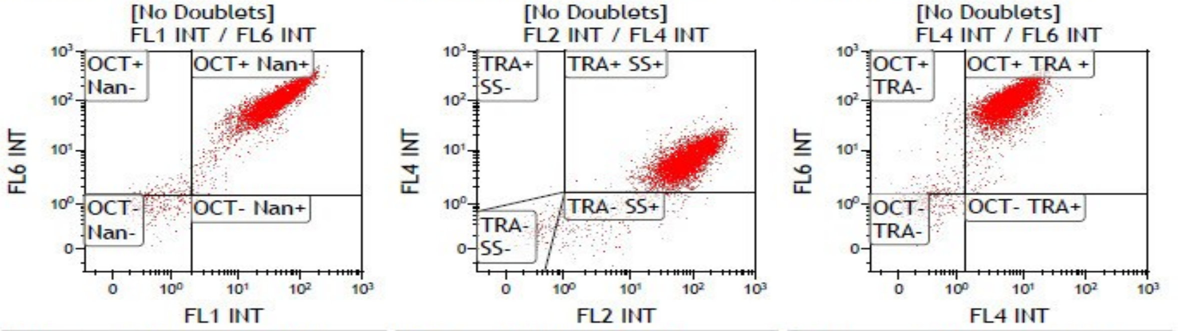

Gate	Number	%Gated
All	8,580	100.00
OCT- Nan-	272	3.17
OCT- Nan+	11	0.13
OCT+ Nan-	111	1.29
OCT+ Nan+	8,186	95.41

Gate	Number	%Gated
All	8,580	100.00
TRA- SS-	149	1.74
TRA- SS+	358	4.17
TRA+ SS-	1	0.01
TRA+ SS+	8,072	94.08

Gate	Number	%Gated
All	8,580	100.00
OCT- TRA-	289	3.37
OCT- TRA+	10	0.12
OCT+ TRA-	170	1.98
OCT+ TRA +	8,111	94.53

iPS Cell Directed Differentiation: Germ Layers

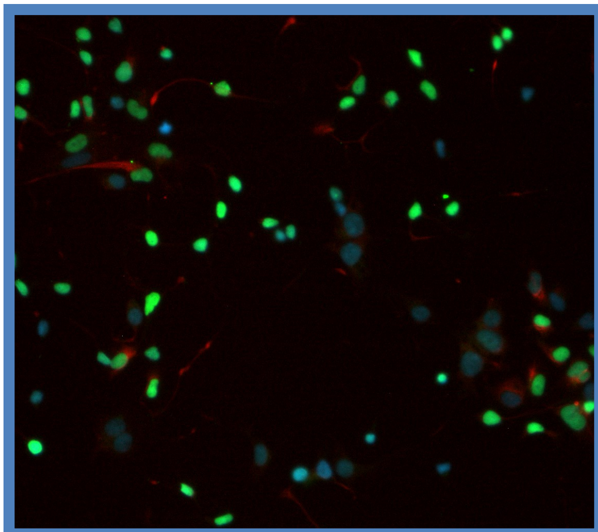

Ectoderm: *iPS cell clone underwent directed differentiation for 6 to 10 days, formalin fixed and Nestin and Pax-6 expression identified by immunohistochemistry. DAPI counterstain and image taken at 40X.*

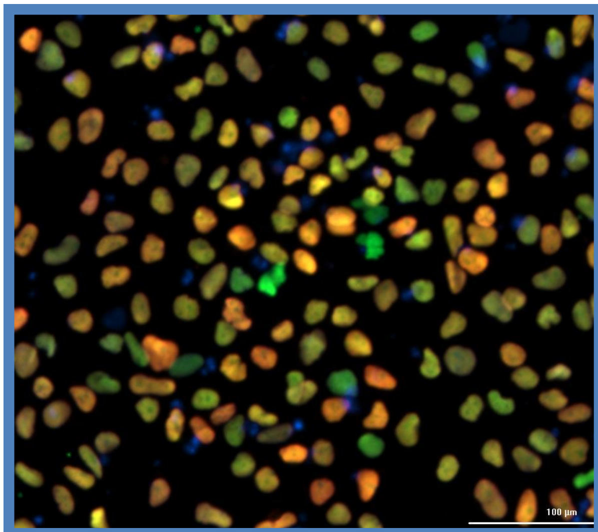

Endoderm: *iPS cell clone underwent directed differentiation for 5 days, formalin fixed and FoxA2 and SOX17 expression identified by immunohistochemistry. DAPI counterstain and image taken at 40X.*

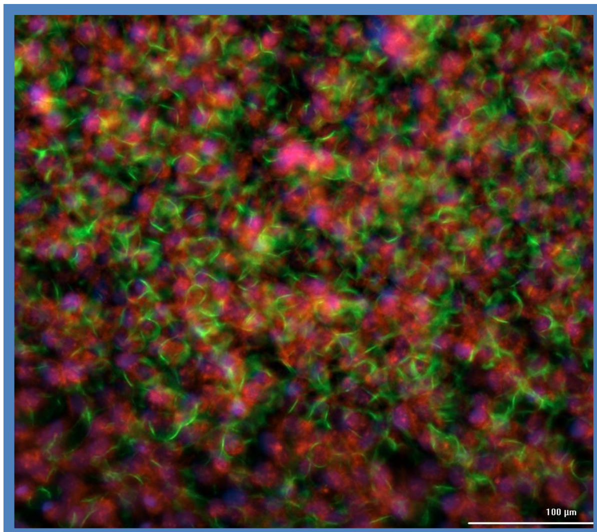

Mesooderm: *iPS cell clone underwent directed differentiation for 14 to 21 days, formalin fixed and CD31 and NCAM expression identified by immunohistochemistry. DAPI counterstain and image taken at 20X.*

Karyotype Analysis

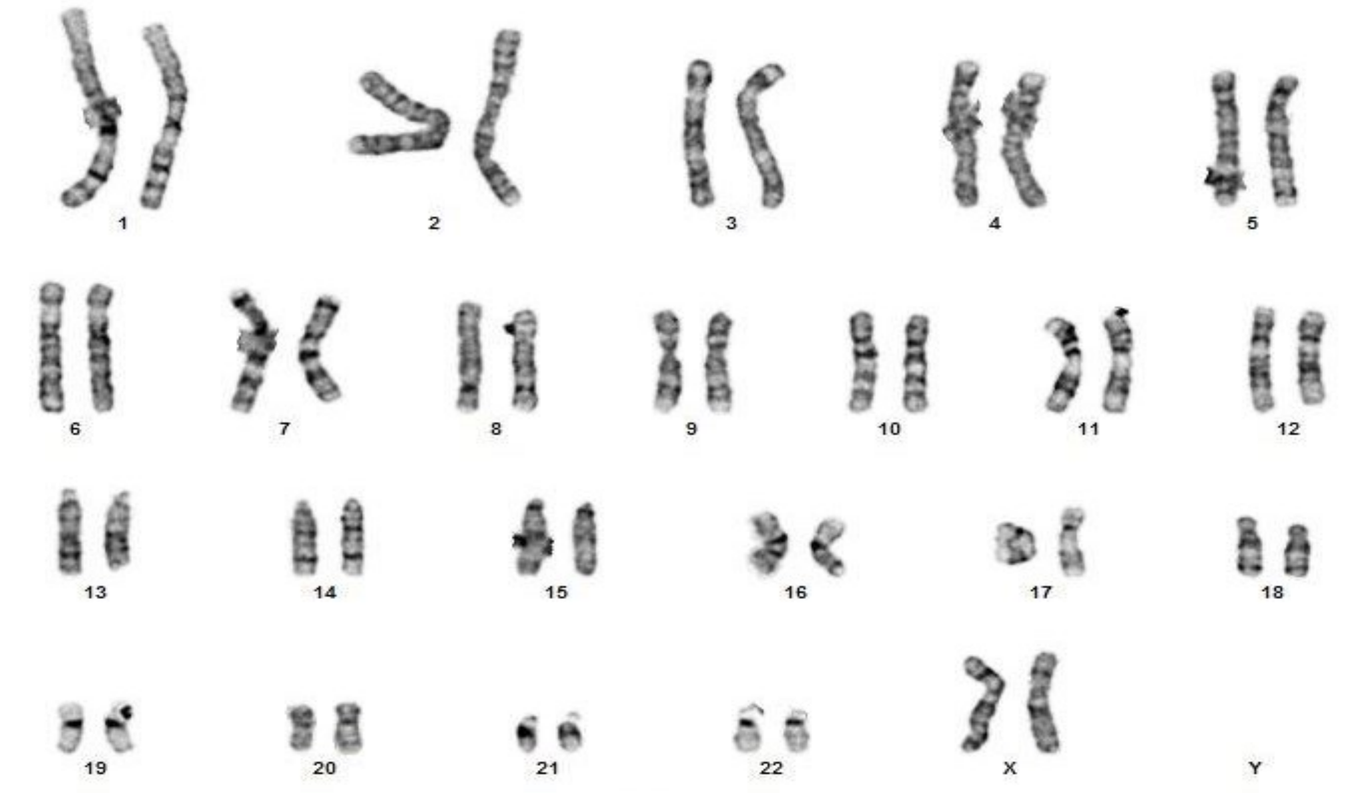

46,XX

MAYO CLINIC

Cytogenetics Laboratory

Mayo Clinic Laboratories-Rochester Main Campus, 200 First Street SW, Rochester, Minnesota 55905

Mayo Comp Cancer Center

Name: Cl. 221 p7 011-BIOTR-0002, , **DOB:** Not provided **Collect Date:** 10/01/2014 10:00AM **Requested By:** Dr. Zachary Resch

Clinic #: **Age:** **Rec'd Date:** 10/01/2014 12:58PM **Location:** Rochester, MN

Accession #: **Gender:** U **Order Date:** 10/01/2014 12:57PM **Source:** hiPSC

Lab ID: 998835 **Specimen:** Cultured cells

REASON FOR REFERRAL

chromosome analysis

METHOD

Tumor culture

BANDING METHOD	CELLS ANALYZED	CELLS COUNTED	CELLS KARYOTYPED	EST. BAND RESOLUTION
GTL	20	0	2	
Total	20	0	2	400

RESULT

46,XX[20]

INTERPRETATION

NO CHARGE

No clonal abnormality was apparent.

Reviewed and approved by Released: 10/31/2014 5:25PM

Patricia Greipp, DO

iPS Cell Pluripotency Marker Expression

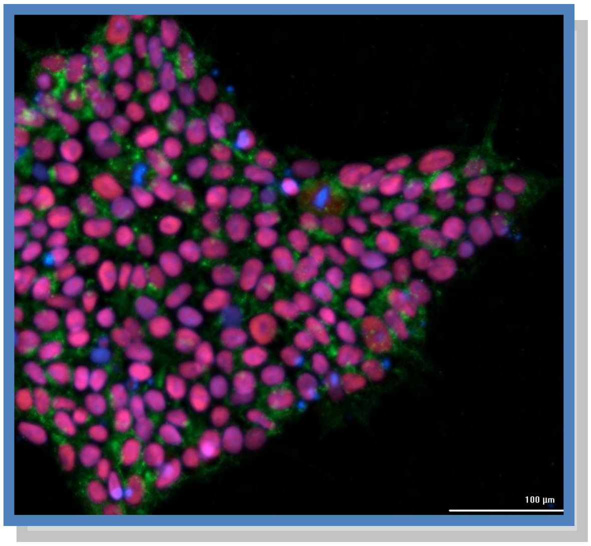

Clone-specific iPS cell colony (Passage ≤ 10) stained for the pluripotency markers Oct4 and SSEA with a nuclear counterstain (DAPI). 20X magnification.

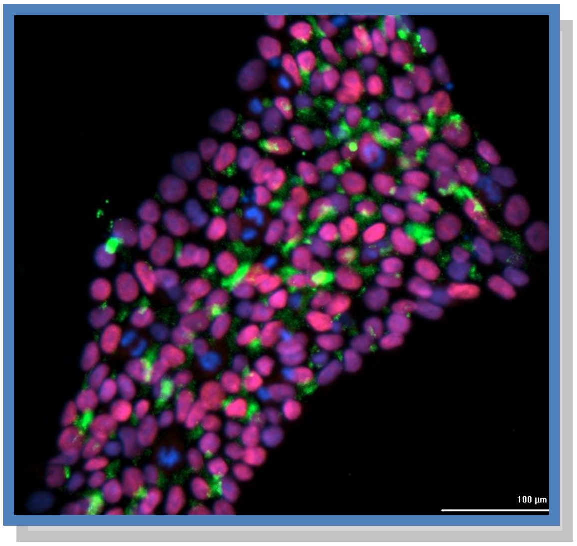

Clone-specific iPS cell colony (Passage ≤ 10) stained for the pluripotency markers Nanog and TRA-1-60 with a nuclear counterstain (DAPI). 20X magnification.

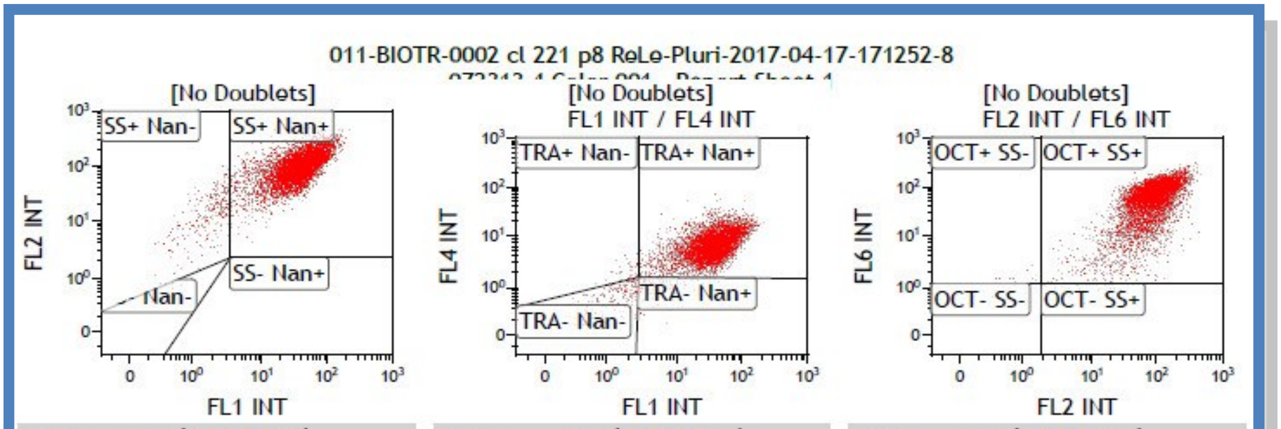

Gate	Number	%Gated
All	7,974	100.00
SS- Nan-	9	0.11
SS- Nan+	0	0.00
SS+ Nan-	259	3.25
SS+ Nan+	7,706	96.64

Gate	Number	%Gated
All	7,974	100.00
TRA- Nan-	167	2.09
TRA- Nan+	110	1.38
TRA+ Nan-	43	0.54
TRA+ Nan+	7,654	95.99

Gate	Number	%Gated
All	7,974	100.00
OCT- SS-	7	0.09
OCT- SS+	37	0.46
OCT+ SS-	9	0.11
OCT+ SS+	7,921	99.34

Gate	Number	%Gated
All	7,974	100.00
OCT- Nan-	27	0.34
OCT- Nan+	3	0.04
OCT+ Nan-	153	1.92
OCT+ Nan+	7,791	97.71

Gate	Number	%Gated
All	7,974	100.00
TRA- SS-	13	0.16
TRA- SS+	263	3.30
TRA+ SS-	1	0.01
TRA+ SS+	7,697	96.53

Gate	Number	%Gated
All	7,974	100.00
OCT- TRA-	40	0.50
OCT- TRA+	11	0.14
OCT+ TRA-	203	2.55
OCT+ TRA +	7,720	96.81

iPS Cell Directed Differentiation: Germ Layers

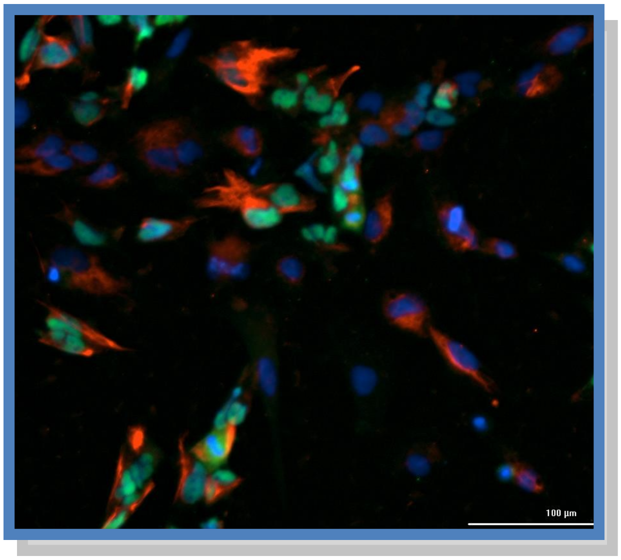

Ectoderm: *iPS cell clone underwent directed differentiation for 6 to 10 days, formalin fixed and Nestin and Pax-6 expression identified by immunohistochemistry. DAPI counterstain and image taken at 20X.*

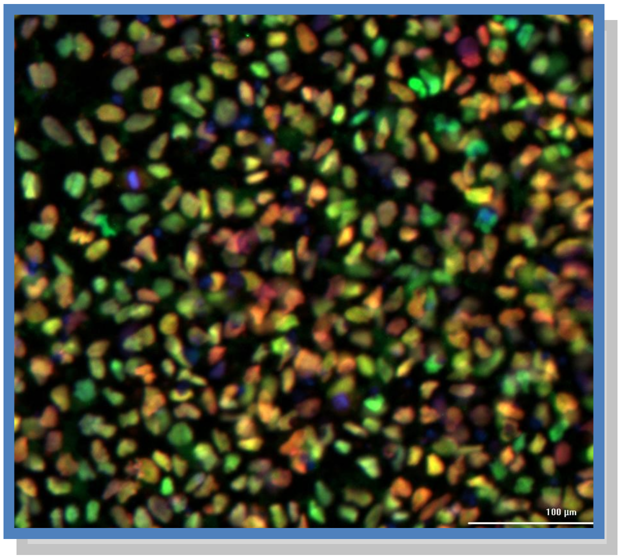

Endoderm: *iPS cell clone underwent directed differentiation for 5 days, formalin fixed and FoxA2 and SOX17 expression identified by immunohistochemistry. DAPI counterstain and image taken at 20X.*

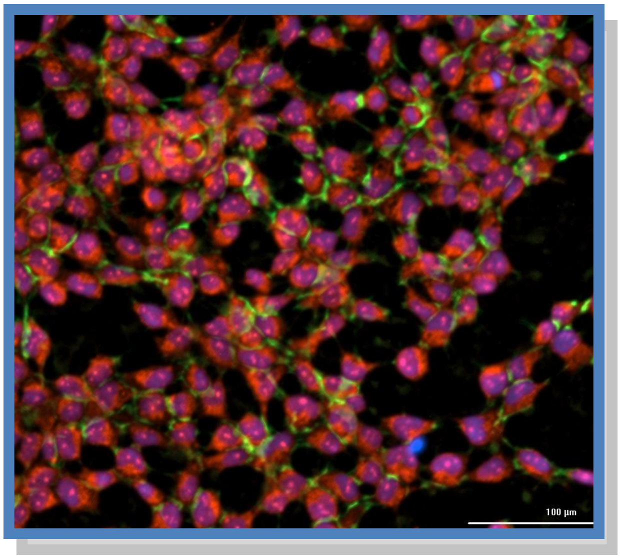

Mesooderm: *iPS cell clone underwent directed differentiation for 5 days, formalin fixed and CD31 and NCAM expression identified by immunohistochemistry. DAPI counterstain and image taken at 20X.*

Karyotype Analysis

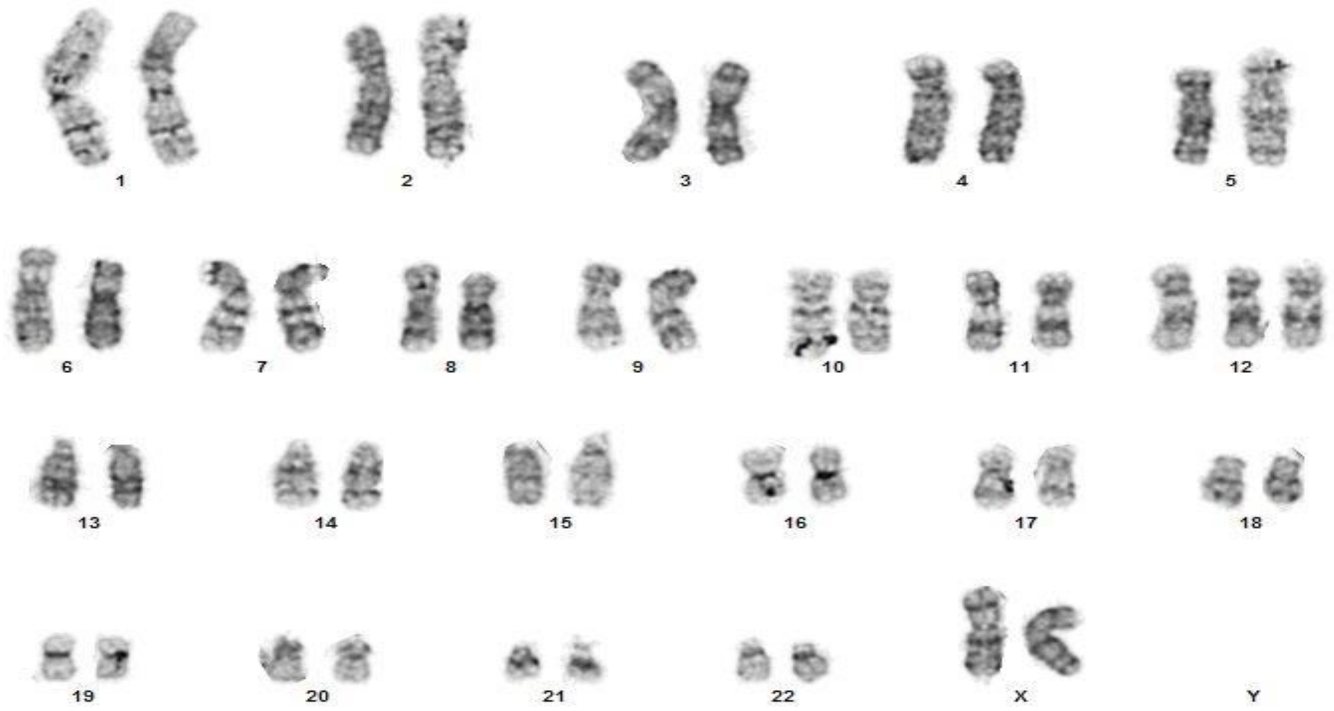

47,XX,+12

MAYO CLINIC
Mayo Medical Laboratories

Performing Site:
Mayo Clinic Laboratories - Rochester Main Campus
200 First Street SW, Rochester MN 55905
William G. Morice, II, M.D., Ph.D. Lab Director
Phone: 800-533-1710
http://www.mayomedicallaboratories.com

011-BIOTR-0003 CL. 17 P18, , MEDICAL RECORD # (PATIENT ID) I128401

DOB	09/09/2015	CLIENT ID/WARD	70000026	ORDER #	9610035940
SEX	Unknown	CLIENT/NAME WARD	Cytogenetics	DATE COLLECTED	9/10/2015 3:06 PM
		CITY, ST, ZIP	ROCHESTER	DATE RECEIVED	9/10/2015 3:06 PM
			MN 55905	DATE REPORTED	11/13/2015 11:48 AM
			REQUESTED BY	ZACHARY T RESCH	

Mayo Clinic Cancer Center

Result Summary:
Abnormal

Interpretation:
The result is abnormal. Of 20 metaphases, 1 was normal and 19 had trisomy 12. This result is consistent with the presence of a clonal abnormality in this specimen of induced pluripotent cells.

Low-level genetic alterations including chromosomal copy number changes (i.e., trisomy, monosomy) of clinical-grade induced pluripotent stem cells and mesenchymal stem cells have been described and may be related to cultural artifact or may be donor dependent. Trisomy 12 is the predominant abnormality recognized in both MSC and iPS cell types. Literature reports that such isolated low-level abnormalities have not typically been associated with a selective growth advantage in vitro and are likely linked to DNA damage-associated senescence (Taapken, et al., Nat Biotechnol 29:313-14, 2011; Tarte et al., Blood 115:1549-1553, 2010; Froelich, et al., Cytotherapy 15:767-781, 2013).

Result:
47,XX,+12[19]/46,XX[1]

iPS Cell Pluripotency Marker Expression

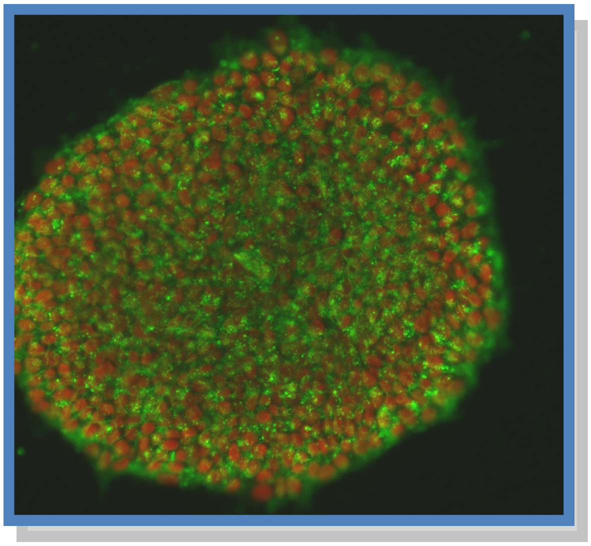

Clone-specific iPS cell colony (Passage ≤ 10) stained for the pluripotency markers Oct4 and SSEA with a nuclear counterstain (DAPI). 20X magnification.

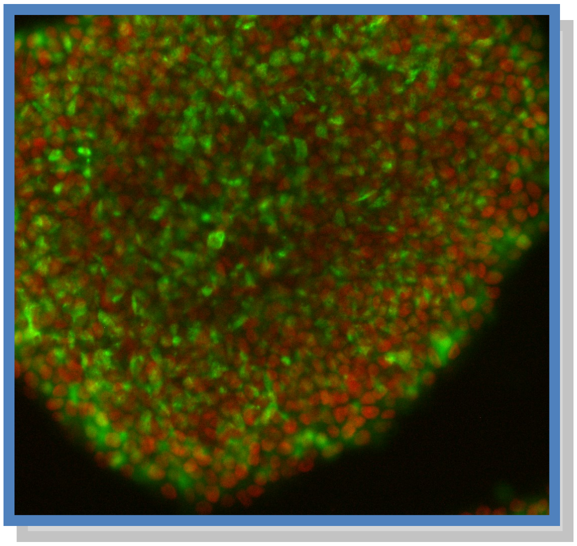

Clone-specific iPS cell colony (Passage ≤ 10) stained for the pluripotency markers Nanog and TRA-1-60 with a nuclear counterstain (DAPI). 20X magnification.

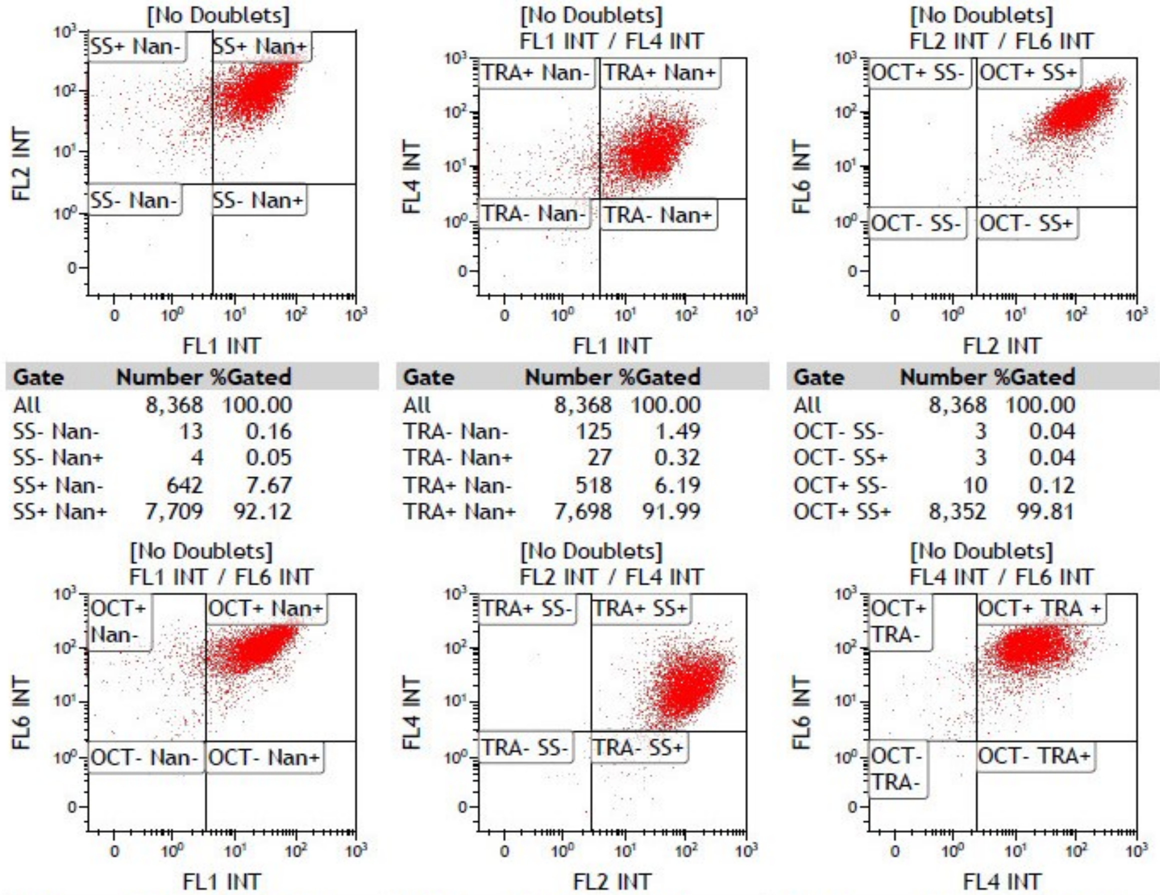

Gate	Number	%Gated
All	8,368	100.00
SS- Nan-	13	0.16
SS- Nan+	4	0.05
SS+ Nan-	642	7.67
SS+ Nan+	7,709	92.12

Gate	Number	%Gated
All	8,368	100.00
TRA- Nan-	125	1.49
TRA- Nan+	27	0.32
TRA+ Nan-	518	6.19
TRA+ Nan+	7,698	91.99

Gate	Number	%Gated
All	8,368	100.00
OCT- SS-	3	0.04
OCT- SS+	3	0.04
OCT+ SS-	10	0.12
OCT+ SS+	8,352	99.81

Gate	Number	%Gated
All	8,368	100.00
OCT- Nan-	6	0.07
OCT- Nan+	0	0.00
OCT+ Nan-	567	6.78
OCT+ Nan+	7,795	93.15

Gate	Number	%Gated
All	8,368	100.00
TRA- SS-	13	0.16
TRA- SS+	161	1.92
TRA+ SS-	3	0.04
TRA+ SS+	8,191	97.88

Gate	Number	%Gated
All	8,368	100.00
OCT- TRA-	9	0.11
OCT- TRA+	0	0.00
OCT+ TRA-	134	1.60
OCT+ TRA +	8,225	98.29

iPS Cell Directed Differentiation: Germ Layers

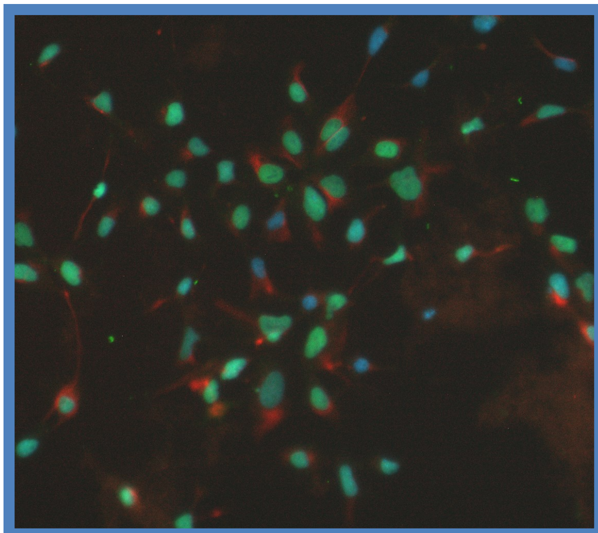

Ectoderm: *iPS cell clone underwent directed differentiation for 6 to 10 days, formalin fixed and Nestin and Pax-6 expression identified by immunohistochemistry. DAPI counterstain and image taken at 20X.*

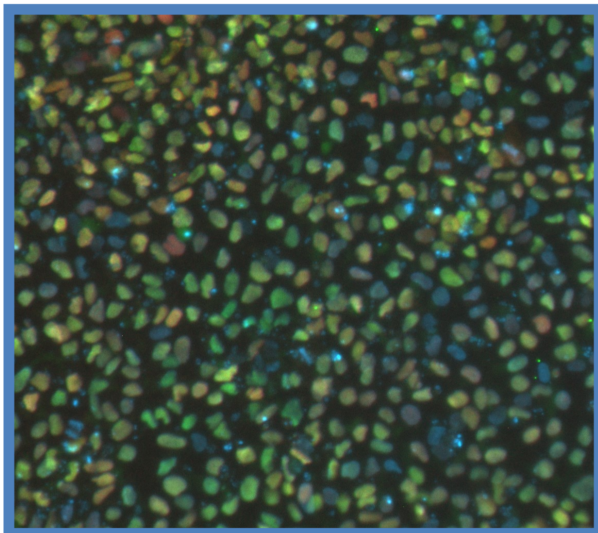

Endoderm: *iPS cell clone underwent directed differentiation for 5 days, formalin fixed and FoxA2 and SOX17 expression identified by immunohistochemistry. DAPI counterstain and image taken at 20X.*

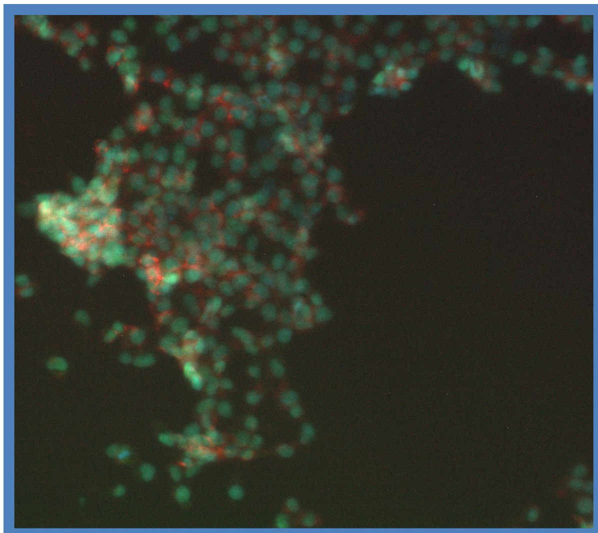

Mesooderm: *iPS cell clone underwent directed differentiation for 5 days, formalin fixed and NCAM and Brachyury expression identified by immunohistochemistry. DAPI counterstain and image taken at 20X.*

Karyotype Analysis

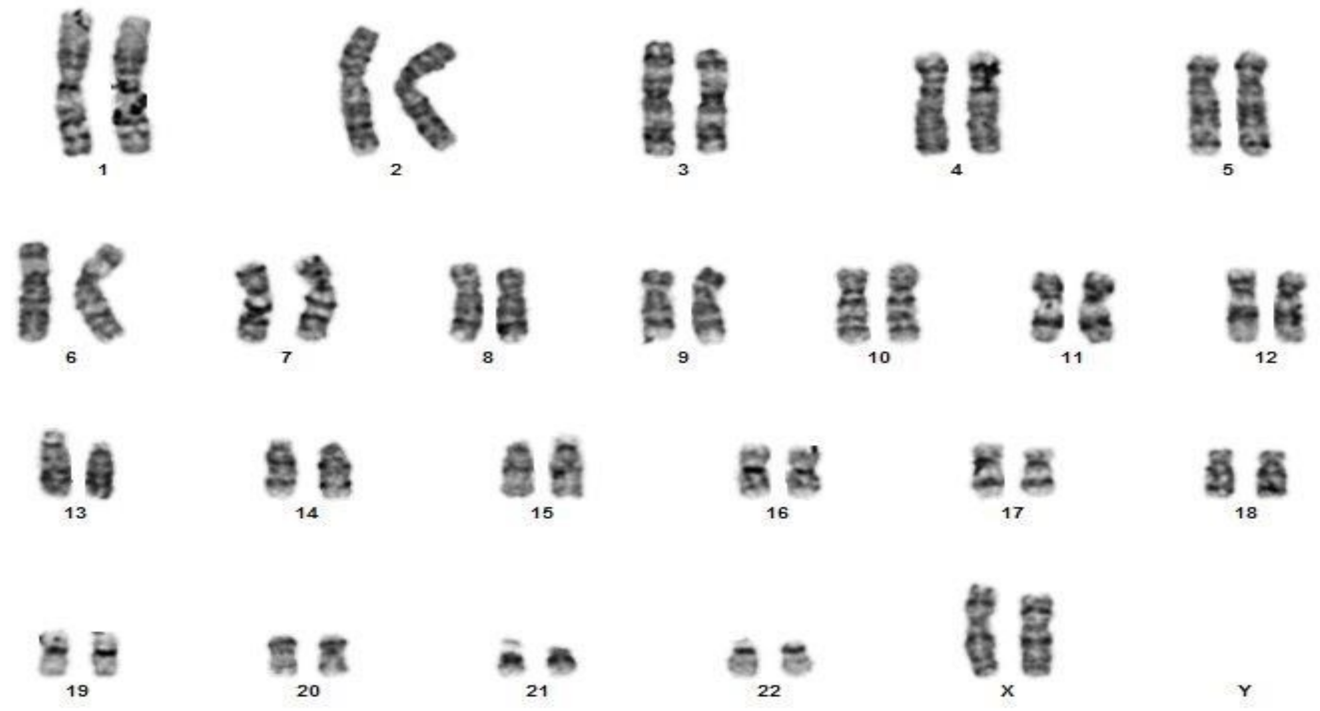

46,XX

MAYO CLINIC

Cytogenetics Laboratory

Mayo Clinic Laboratories-Rochester Main Campus, 200 First Street SW, Rochester, Minnesota 55905

Mayo Comp Cancer Center

Name: 011BIOTR0003Cl.226p7, , **DOB:** Not provided **Collect Date:** 09/02/2014 12:00AM **Requested By:** Dr. Zachary Resch

Clinic #: **Age:** **Rec'd Date:** 09/02/2014 12:03PM **Location:** Rochester, MN

Accession #: **Gender:** U **Order Date:** 09/02/2014 11:58AM **Source:** cultured cells

Lab ID: 992825 **Specimen:** Cultured cells

REASON FOR REFERRAL

chromosome analysis

METHOD

Tumor culture

BANDING METHOD	CELLS ANALYZED	CELLS COUNTED	CELLS KARYOTYPED	EST. BAND RESOLUTION
GTL	20	0	2	
Total	20	0	2	400

RESULT

46,XX[20]

INTERPRETATION

NO CHARGE

No clonal abnormality was apparent.

Reviewed and approved by

Released: 09/10/2014 3:42PM

Patricia Greipp, DO

iPS Cell Pluripotency Marker Expression

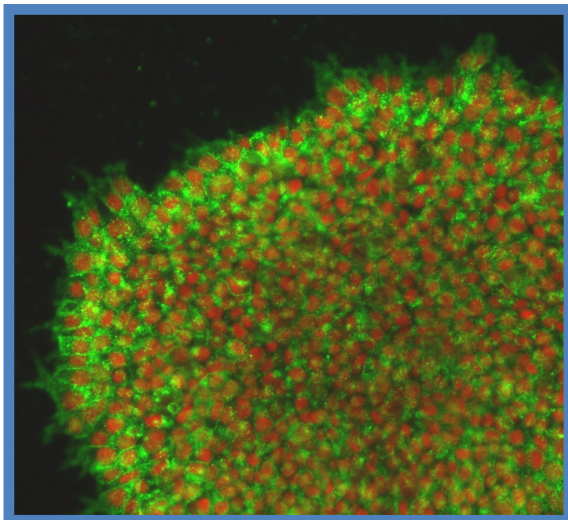

Clone-specific iPS cell colony (Passage < 10) stained for the pluripotency markers Oct4 and SSEA with a nuclear counterstain (DAPI). 20X magnification.

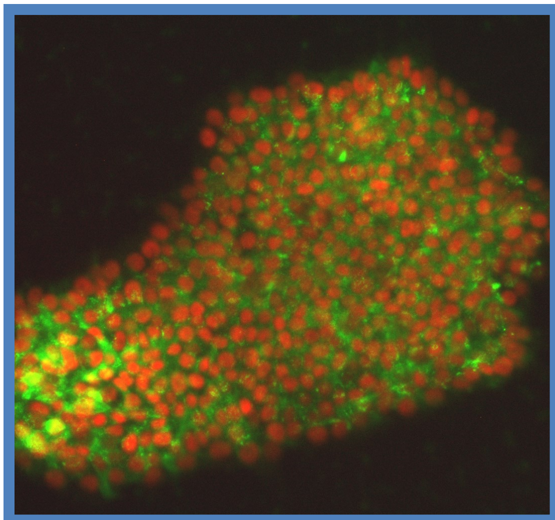

Clone-specific iPS cell colony (Passage < 10) stained for the pluripotency markers Nanog and TRA-1-60 with a nuclear counterstain (DAPI). 20X magnification.

011-BIOTR-0003cl226 Stained 002
072313 4 Color 001 - Report Sheet 1

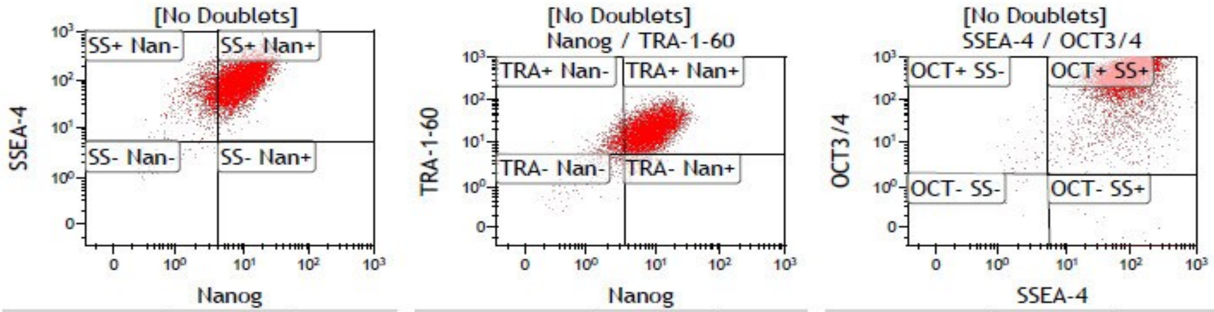

Gate	Number	%Gated
All	8,197	100.00
SS- Nan-	47	0.57
SS- Nan+	2	0.02
SS+ Nan-	1,171	14.29
SS+ Nan+	6,977	85.12

Gate	Number	%Gated
All	8,197	100.00
TRA- Nan-	245	2.99
TRA- Nan+	275	3.35
TRA+ Nan-	519	6.33
TRA+ Nan+	7,158	87.32

Gate	Number	%Gated
All	8,197	100.00
OCT- SS-	9	0.11
OCT- SS+	252	3.07
OCT+ SS-	41	0.50
OCT+ SS+	7,895	96.32

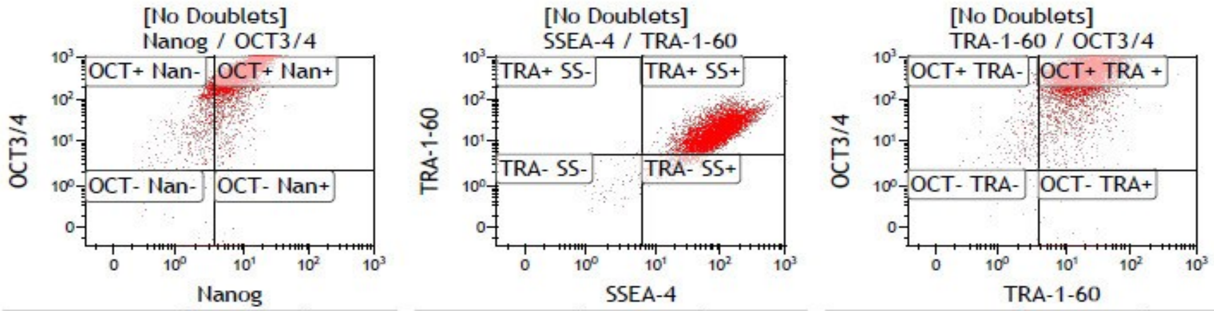

Gate	Number	%Gated
All	8,197	100.00
OCT- Nan-	173	2.11
OCT- Nan+	92	1.12
OCT+ Nan-	764	9.32
OCT+ Nan+	7,168	87.45

Gate	Number	%Gated
All	8,197	100.00
TRA- SS-	56	0.68
TRA- SS+	464	5.66
TRA+ SS-	3	0.04
TRA+ SS+	7,674	93.62

Gate	Number	%Gated
All	8,197	100.00
OCT- TRA-	30	0.37
OCT- TRA+	237	2.89
OCT+ TRA-	212	2.59
OCT+ TRA +	7,718	94.16

iPS Cell Directed Differentiation: Germ Layers

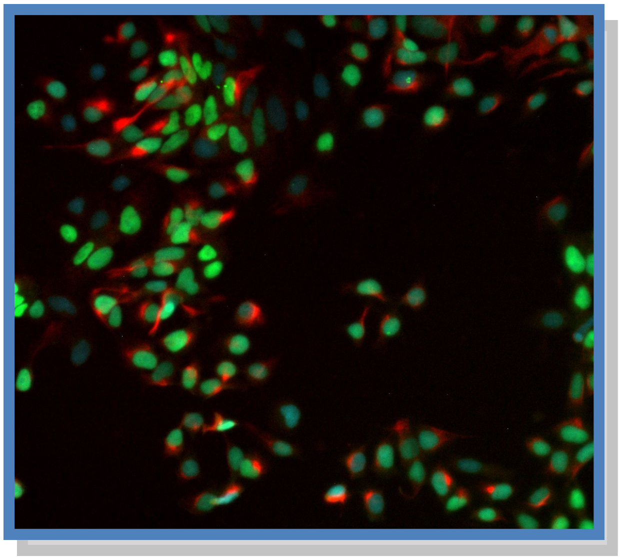

Ectoderm: *iPS cell clone underwent directed differentiation for 6 to 10 days, formalin fixed and Nestin and Pax-6 expression identified by immunohistochemistry. DAPI counterstain and image taken at 20X.*

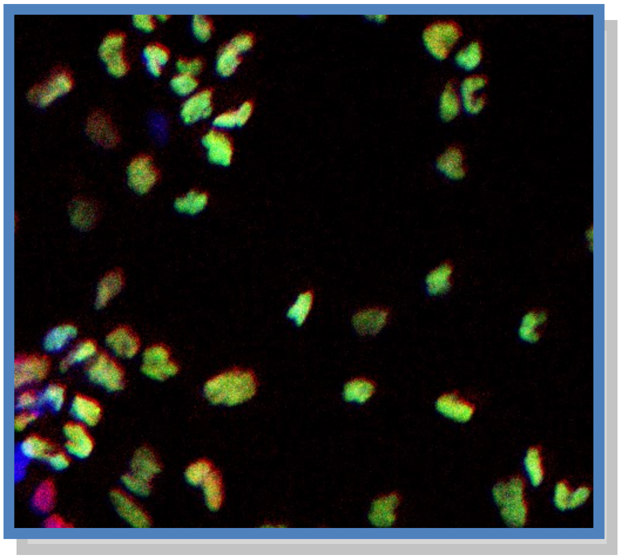

Endoderm: *iPS cell clone underwent directed differentiation for 5 days, formalin fixed and FoxA2 and SOX17 expression identified by immunohistochemistry. DAPI counterstain and image taken at 40X.*

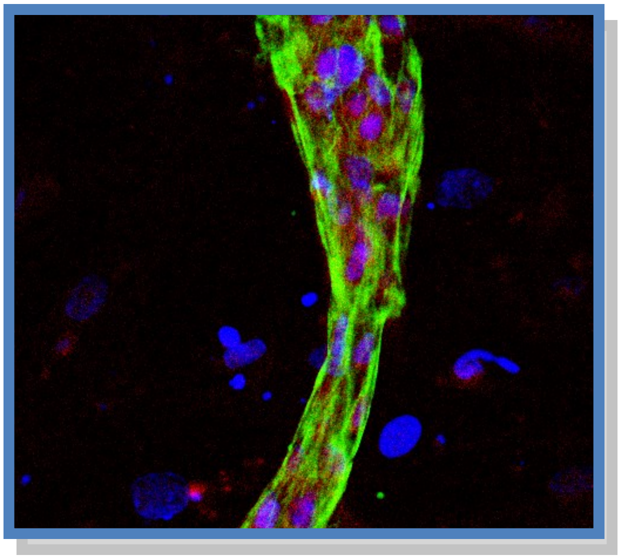

Mesooderm: *iPS cell clone underwent directed differentiation for 14 to 21 days, formalin fixed and NKX2.5 and TNNT expression identified by immunohistochemistry. DAPI counterstain and image taken at 40X.*

Karyotype Analysis

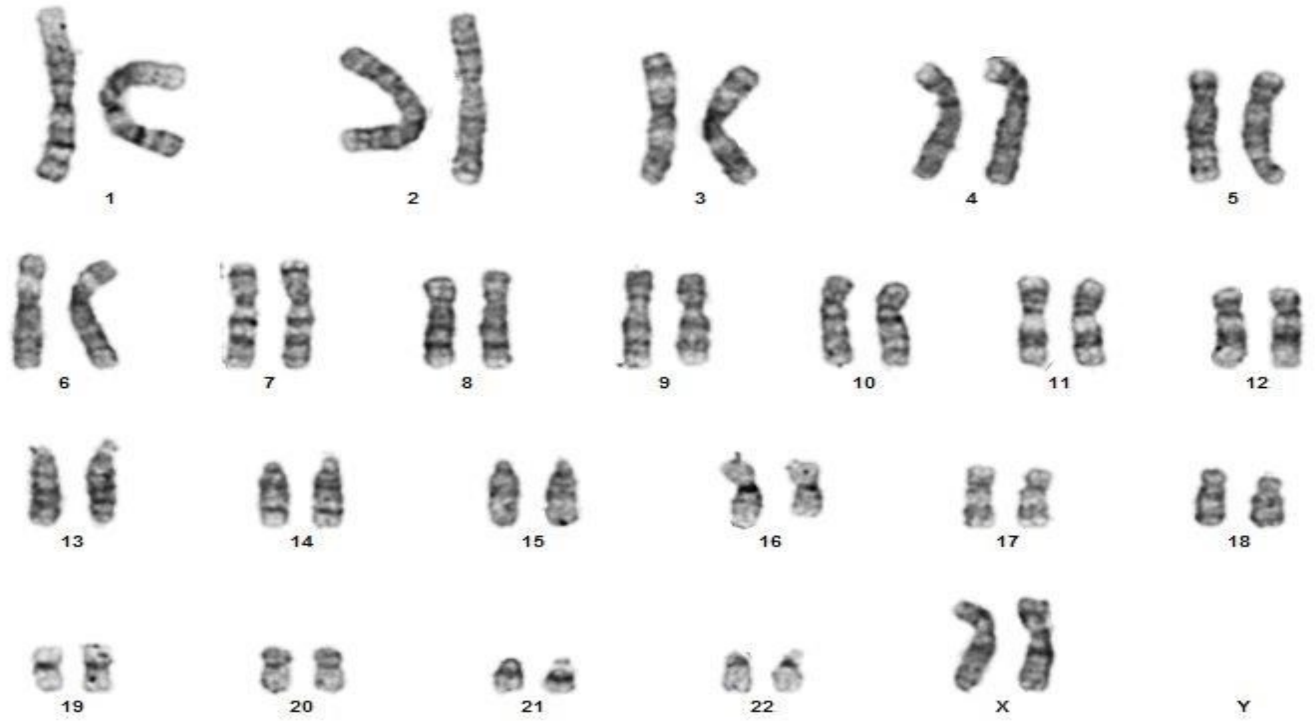

46,XX

MAYO CLINIC

Cytogenetics Laboratory
Mayo Clinic Laboratories-Rochester Main Campus, 200 First Street SW, Rochester, Minnesota 55905

Mayo Comp Cancer Center

**Name:** Cl. 243 p10 011-BIOTR-0003,	**DOB:** Not provided	**Collect Date:** 09/23/2014 10:00AM	**Requested By:** Dr. Zachary Resch
**Clinic #:**	**Age:**	**Rec'd Date:** 09/23/2014 2:13PM	**Location:** Rochester, MN
**Accession #:**	**Gender:** U	**Order Date:** 09/23/2014 2:12PM	**Source:** hiPSC
**Lab ID:** 997142	**Specimen:** Cultured cells		

REASON FOR REFERRAL

chromosome analysis

METHOD

Tumor culture

BANDING METHOD	CELLS ANALYZED	CELLS COUNTED	CELLS KARYOTYPED	EST. BAND RESOLUTION
GTL	20	0	2	
Total	20	0	2	400

RESULT

46,XX[20]

INTERPRETATION

NO CHARGE

No clonal abnormality was apparent.

Reviewed and approved by

Released: 11/04/2014 1:45PM

Patricia Greipp, DO

iPS Cell Pluripotency Marker Expression

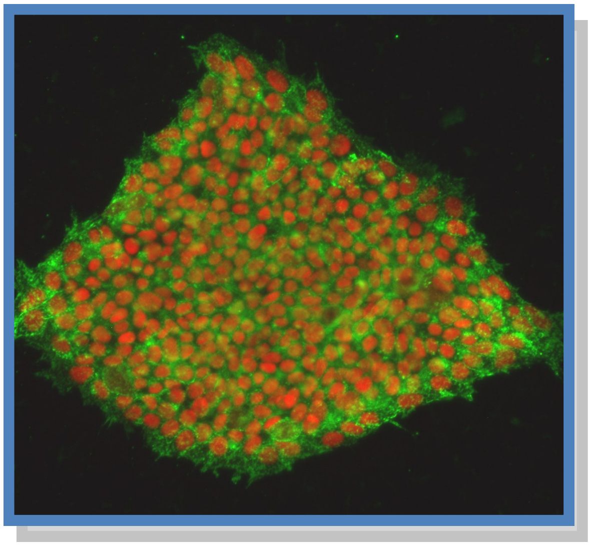

Clone-specific iPS cell colony (Passage ≤ 10) stained for the pluripotency markers Oct4 and SSEA with a nuclear counterstain (DAPI). 20X magnification.

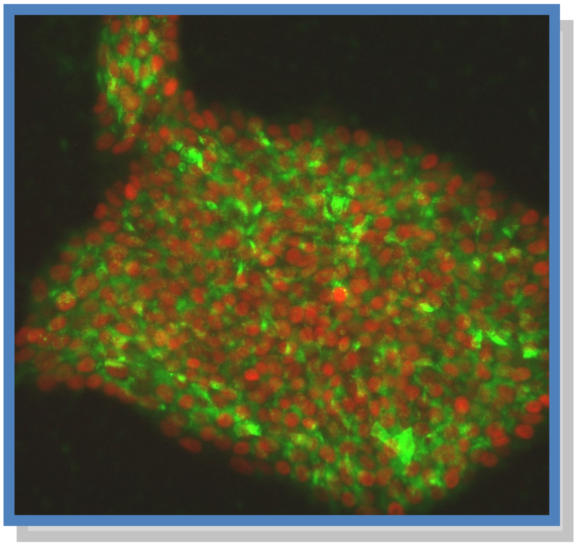

Clone-specific iPS cell colony (Passage ≤ 10) stained for the pluripotency markers Nanog and TRA-1-60 with a nuclear counterstain (DAPI). 20X magnification.

011-BIOTR-0003cl243 Stained 004
072313 4 Color 001 - Report Sheet 1

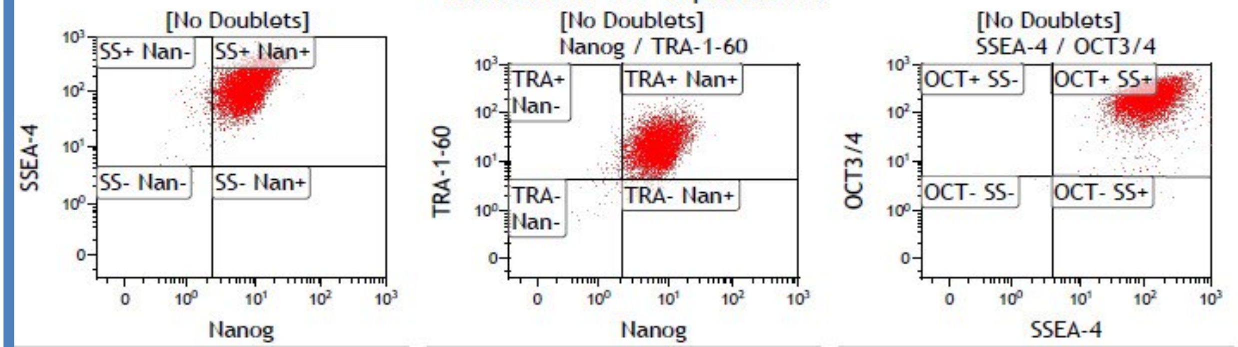

Gate	Number	%Gated
All	7,486	100.00
SS- Nan-	2	0.03
SS- Nan+	0	0.00
SS+ Nan-	126	1.68
SS+ Nan+	7,358	98.29

Gate	Number	%Gated
All	7,486	100.00
TRA- Nan-	25	0.33
TRA- Nan+	71	0.95
TRA+ Nan-	63	0.84
TRA+ Nan+	7,327	97.88

Gate	Number	%Gated
All	7,486	100.00
OCT- SS-	2	0.03
OCT- SS+	14	0.19
OCT+ SS-	0	0.00
OCT+ SS+	7,470	99.79

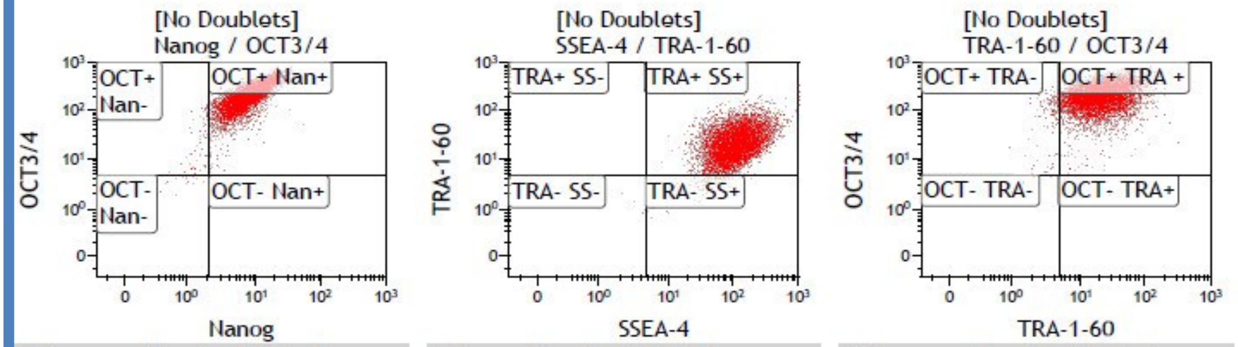

Gate	Number	%Gated
All	7,486	100.00
OCT- Nan-	14	0.19
OCT- Nan+	2	0.03
OCT+ Nan-	72	0.96
OCT+ Nan+	7,398	98.82

Gate	Number	%Gated
All	7,486	100.00
TRA- SS-	4	0.05
TRA- SS+	127	1.70
TRA+ SS-	0	0.00
TRA+ SS+	7,355	98.25

Gate	Number	%Gated
All	7,486	100.00
OCT- TRA-	9	0.12
OCT- TRA+	7	0.09
OCT+ TRA-	173	2.31
OCT+ TRA +	7,297	97.48

iPS Cell Directed Differentiation: Germ Layers

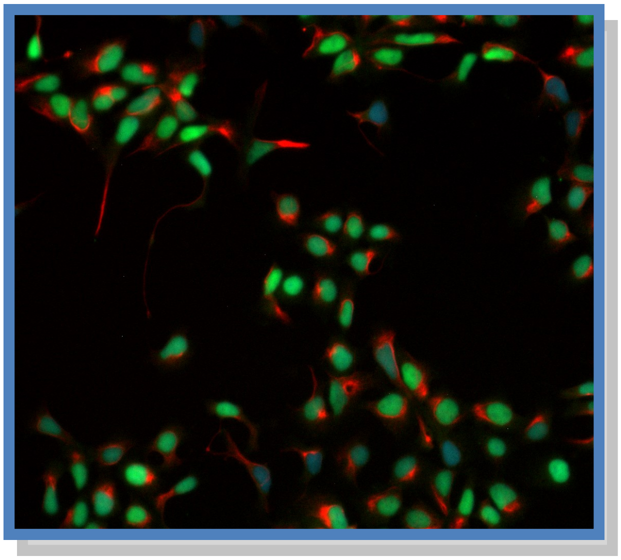

Ectoderm: *iPS cell clone underwent directed differentiation for 6 to 10 days, formalin fixed and Nestin and Pax-6 expression identified by immunohistochemistry. DAPI counterstain and image taken at 20X.*

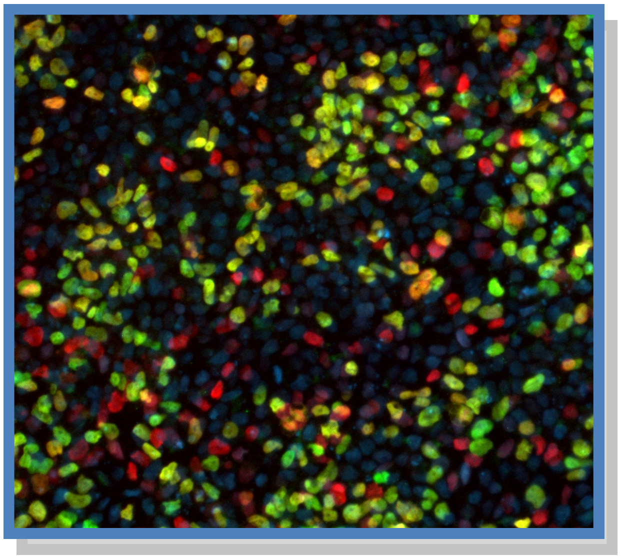

Endoderm: *iPS cell clone underwent directed differentiation for 5 days, formalin fixed and FoxA2 and SOX17 expression identified by immunohistochemistry. DAPI counterstain and image taken at 20X.*

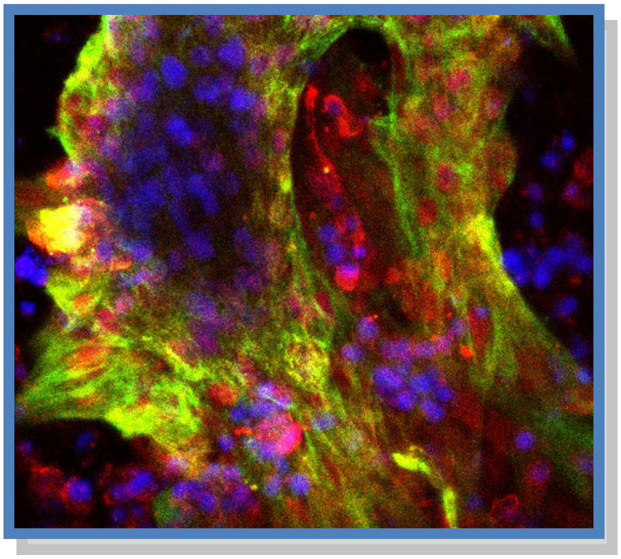

Mesooderm: *iPS cell clone underwent directed differentiation for 14 to 21 days, formalin fixed and NKX2.5 and TNNT expression identified by immunohistochemistry. DAPI counterstain and image taken at 40X.*
